# Associations between dietary macronutrient composition and cardiometabolic health: data from NHANES 1999–2014

**DOI:** 10.1007/s00394-024-03523-7

**Published:** 2024-12-07

**Authors:** Nicholas A. Koemel, Alistair M. Senior, Nasser Laouali, David S. Celermajer, Amanda Grech, Helen M. Parker, Stephen J. Simpson, David Raubenheimer, Timothy P. Gill, Michael R. Skilton

**Affiliations:** 1 Charles Perkins Centre, The University of Sydney, Level 2, John Hopkins Drive, Sydney, NSW 2006 Australia; 2 Sydney Medical School, The University of Sydney, Sydney, Australia; 3 School of Life and Environmental Sciences, The University of Sydney, Sydney, Australia; 4 Université Paris-Saclay, CESP UMR1018, UVSQ, Inserm, Gustave Roussy, Villejuif, Paris, France; 5 Department of Biostatistics and Epidemiology, School of Public Health and Health Sciences, University of Massachusetts, Amherst, MA USA; 6 Scripps Institution of Oceanography, University of California, San Diego, US; 7 Susan Wakil School of Nursing and Midwifery, The University of Sydney, Sydney, Australia

**Keywords:** NHANES, Diet, Macronutrients, Cardiometabolic health, Cardiovascular disease

## Abstract

**Purpose:**

Dietary macronutrients significantly impact cardiometabolic health, yet research often focuses on individual macronutrient relationships. This study aimed to explore the associations between dietary macronutrient composition and cardiometabolic health.

**Methods:**

This study included 33,681 US adults (49.7 ± 18.3 years; 52.5% female) from the National Health and Nutrition Examination Survey during 1999–2014. Dietary data was derived from 1 to 2 separate 24-hour recalls and cardiometabolic health included lipid profile, glycemic control, blood pressure, and adiposity collected in a mobile examination center. Associations between dietary macronutrient composition and cardiometabolic health were examined using generalized additive models adjusted for age, socio-demographics, lifestyle, and diet quality.

**Results:**

In females, triglycerides (*P* < 0.01) and HDL cholesterol (*P* < 0.01) were the least optimal in diets containing lower fat (10%) and higher carbohydrate (75%). In males, HDL cholesterol was positively associated with fat (*P* < 0.01) and no association with triglycerides was detected. Total-C associations were male specific (*P* = 0.01) and highest in diets composed of 25% protein, 30% carbohydrate, and 45% fat. In both sexes, systolic blood pressure (*P* ≤ 0.02) was highest in diets containing lower fat (10%) coupled with moderate protein (25%). Diastolic blood pressure associations were female specific (*P* < 0.01) with higher values in those consuming the upper range of fat (55%). There were no associations of macronutrient composition with glycemic control or adiposity.

**Conclusion:**

This study revealed sex-specific relationships between macronutrient composition and cardiometabolic health. Future research is needed to explore these relationships across age groups.

**Supplementary Information:**

The online version contains supplementary material available at 10.1007/s00394-024-03523-7.

## Introduction

Noncommunicable diseases (NCDs) account for 71% of all deaths globally, of which cardiovascular disease (CVD) and cancer are the primary contributors [1]. Diet and lifestyle play an essential role in the prevention and treatment of NCDs [2]. This appears to be partially mediated by associations of diet with cardiovascular and metabolic risk factors such as blood lipid profile, glycemic regulation, blood pressure, and adiposity [3]. Thus, improved nutrition presents a key target for reducing the global burden of NCDs.

Nutritional epidemiology has traditionally focused on the role of individual nutrients in human health [4]. However, this does not characterize potential interactive associations between nutrient components such as macronutrients. Both observational and experimental research frequently focus on singular dietary macronutrients, such as high-carb or high-protein diets. This narrow scope poses challenges in identifying the broader impact of macronutrient composition [5, 6]. Moreover, focusing on individual macronutrients has led to a wide degree of controversy regarding the potential therapeutic and detrimental effects of macronutrients [7]. One example of this can be seen with unique tradeoffs that have been observed for cardiometabolic health across diets of varying macronutrient composition [8–13]. For example, diets high in dietary fat have been shown to have a beneficial effect on satiety and HDL cholesterol while worsening glycemic control and fasting triglycerides [7, 8, 14]. While exploring whole foods or dietary patterns may address a proportion of these interactions [15], it cannot precisely isolate the overall influence of specific nutrients nor partition their effects on these outcomes.

The Geometric Framework for Nutrition (GFN) is a multidimensional approach utilized to better capture the complex nature of nutrition [16]. The GFN uses state-space response surfaces to visualize non-linear interactive associations with outcomes of interest [17]. This technique has been widely used in animal and human models for understanding the link between macronutrient composition, cardiometabolic health, noncommunicable diseases, and lifespan [18–25]. These studies identify distinct interactive effects of dietary macronutrients with cardiometabolic health that are not fully captured by exploring individual macronutrients. However, this technique has yet to be broadly applied to understand the relationship of diet composition with cardiometabolic health from a population-level perspective.

Therefore, we sought to determine the associations of dietary macronutrient composition with cardiometabolic health among US adults. As a secondary aim, we explored the relationship between macronutrient composition with the varying components of the Healthy Eating Index (HEI) to create a more comprehensive understanding of how dietary components and food groups may partially explain the relationship between macronutrients and cardiometabolic health. We hypothesized that the associations of macronutrient profiles would be complex involving trade-offs across varying cardiometabolic health markers.

## Subjects and methods

### Study population

The National Health and Nutrition Examination Survey (NHANES) is an ongoing research program conducted by the National Center for Health Statistics and the Centers for Disease Control and Prevention. NHANES focuses on the nutrition and health of individuals in the United States. This study evaluated male and female participants aged 20 years or older from 1999 to 2014. Dietary data was collected via 1–2 separate 24-hour dietary recalls recorded by a trained nutrition professional. The first recall was collected in-person followed by a second interview that was conducted by telephone 3–10 days later [26]. During the years 1999–2002, only a single 24-hour dietary recall was collected at the initial examination. To derive corresponding nutrient daily nutrient intake, the individual foods and meals identified in the 24-hour dietary recall were processed using a computer-assisted food coding and data management system developed by the United States Department of Agriculture [27, 28]. Individuals with potentially unreliable dietary intake (males with energy intake of < 800 or > 4200 kcal/day or females with an intake of < 600 or > 3500 kcal/day [29]) or macronutrient intakes 3 standard deviations from the mean were not included in the analysis. Participants with a minimum of two 24-hour recalls were adjusted using a validated multiple system method to reflect the habitual intake of nutrients [22, 30–32]. All participants provided informed consent and the Institution Review Board for the CDC approved data collection and public use of all data.

### Cardiometabolic health

NHANES participants were randomly selected for collection of a fasting venous blood sample to determine their serum glucose, insulin, HbA1c, total cholesterol (Total-C), low-density lipoprotein cholesterol (LDL), high-density lipoprotein-cholesterol (HDL), and triglycerides. In addition, a random subset of participants underwent an oral glucose tolerance test (OGTT) where they were given a calibrated dose of glucose drink (TrutolTM, Thermo Scientific, Waltham, MA) providing an average of 75 g of glucose. Postprandial glucose levels were measured 2 hours after consuming the glucose drink. Body fat percentage was estimated via Bio-electrical impedance analysis (HYDRA, Bio-Impedance Spectrum Analyzer, Model 4200, San Diego, CA) which sends a small alternating current through surface electrodes placed on the right hand and foot. Systolic and diastolic blood pressure were measured 3–4 times via sphygmomanometer and their average was used for the study. Trained professionals collected height and weight using standardized operating procedures, and body mass index (BMI) was calculated as weight divided by height in meters squared (kg/m^2^). Please refer to the NHANES page for information on questionnaires and laboratory procedures for further details on the methods employed [27].

### Socio-demographic and lifestyle covariates

Participants self-reported their demographic and lifestyle characteristics. Race/ethnicity information was used to categorize individuals as non-Hispanic white, non-Hispanic black, Hispanic, or other. Education was grouped into three categories: less than high school, high school, or some college and above. Smokers were defined as individuals who reported smoking more than 100 cigarettes during their lifetime, while alcohol consumers were those who had consumed a minimum of 12 drinks in a year. Physical activity was quantified based on the self-reported metabolic equivalents (METs) of moderate to vigorous leisure activities completed each week. Dietary quality was assessed using the 2015 HEI, which measures adherence to the 2015–2020 US Dietary Guidelines, and assigns a score ranging from 0 to 100 points [33]. The 2015 HEI measures the intake of various food groups to ensure adequate consumption of fruits, vegetables, beans, grains, dairy, protein foods, seafood, and plant protein, while also promoting a healthy balance of polyunsaturated (PUFA) and monounsaturated (MUFA) fatty acids to saturated fatty acids (SFA) defined at the fatty acid ratio ((PUFA + MUFA/SFA)). Additionally, the HEI includes a moderation category that takes into account consumption of refined grains, sodium, added sugars, and saturated fat (Supplementary Table 1). During 1999–2002, the HEI score calculation included a moderation component known as empty calories, which includes the percentage of total dietary energy from solid fats, alcohol, and added sugars.

### Statistical analysis

The relationship between dietary macronutrients and cardiometabolic health was analyzed using generalized additive models (GAMs). GAMs are a form of multivariable regression that enable both visual and statistical assessment of nonlinear associations [34, 35]. GAMs fit continuous variables using a flexible smoothing function that can conform to both linear and complex nonlinear relationships. Similar to other forms of regression, GAMs also allow for continuous and categorical covariate adjustment as seen in traditional epidemiological literature. In the present study, three separate models were created to sequentially adjust for covariates. Model one was the base model and included adjustment for age and household income. Model two was adjusted as per model one, with further adjustment for socio-demographic characteristics, specifically ethnicity and education. Model three was adjusted as per model two, with further adjustment for lifestyle habits including alcohol intake, smoking, physical activity, BMI, and HEI. To account for potential differences in food selection and metabolism we explored all models stratified by sex. All GAMs were constructed using the ‘gam’ function from the *mgcv* package in R, estimated by generalized-cross validation (GCV) score, and checked for concurvity or overfitting (v. 1.8–41; R Core Team; Vienna, Austria). The *mgcv* package does not enable the use of survey weights, thus these results are not fully representative of the US population.

A transformational framework was used to visualize dietary associations with our primary outcomes. Here, GAMs modeled the absolute intake of all macronutrients (i.e., protein; carbohydrate; fat) simultaneously as 3-dimensional smooth term to predict each outcome of cardiometabolic health. Absolute intake of macronutrients (kcal per day) were then transformed to reflect a proportion of total macronutrient energy where outcomes were plotted as a response surface on a mixture triangle using the *ggplot2* package in R statistical software (v. 3.3.6; R Core Team; Vienna, Austria) [36]. Macronutrient composition is shown on a mixture triangle where the *x* and *y-axis* represent the percentage of dietary protein and carbohydrates [37]. Fat intake can be inferred by subtracting the sum of protein and carbohydrate from 100, such that every point on a triangle will sum to 100. Compositional associations were predicted at the sex-specific 50th percentile of total dietary energy intake from all sources. Each outcome is presented on the surface as an RGB spectrum, where warm colors represent higher values and cool colors represent lower values, with the addition of contour lines that contain numeric values of the specified outcome. To avoid over-extrapolation of each model, the response surface for each outcome only presents predictions across the dietary macronutrient ranges observed in this dataset. Cardiometabolic markers were log-transformed with predicted values back-transformed onto the original scale. All thirteen components of the HEI were plotted as individual response surfaces at the 50th percentile of energy intake and displayed in the Supplementary Material. All other statistical analyses were undertaken with SPSS (Version 27; IBM Corporation; Armonk, NY). Statistical significance was declared at *P* < 0.05.

### Additional and sensitivity analyses

To account for possible differences in dietary macronutrient compositions across varying energy intakes, we provide extended response surfaces plotted at the 25th, 50th, and 75th sex-specific percentile of total energy intake. Additional analyses were conducted to examine compositional associations stratified by BMI (above and below 25) and age (above and below the sex-specific median age). A pregnancy sensitivity was conducted by removing those reported as pregnant in the female stratified analysis. We also completed a sensitivity analysis excluding individuals who reported having hypertension, type two diabetes, cardiovascular disease, cancer, or were recommended by a doctor to take anti-hypertensive, lipid-lowering, or glucose controlling medication. Lastly, a sensitivity analysis was completed excluding individuals who did not have two completed 24-hour recalls.

## Results

### Participant characteristics

Participant demographic and cardiometabolic characteristics for the individuals included in this analysis are shown in Table 1. This sample included 33,681 US adults (49.7 ± 18.3 years; 52.5% female; Supplementary Fig. 1). Of these participants, pregnant women were included in the primary analysis (*n* = 1,008). At the time of the interview, reported comorbidities included diabetes (*n* = 3,826), cardiovascular disease (*n* = 3,384), and cancer (*n* = 3,068). Self-reported medication for related comorbid conditions included that for diabetes (*n* = 2,831), hypertension (*n* = 7,107), and lipid-lowering medications (*n* = 4,325).

**Table 1:** Participant Characteristics

Participant Characteristics	Mean	SD
Age (years)	49.7	18.4
Female sex (n%)	52.5	−
BMI (kg/m^2^)	28.8	6.5
Total Energy (kcal)	1,907	603
Healthy Eating Index Score	52.7	12.8
Protein (kcal)	290	106
Protein (TEI%)	15.2	4.0
Carbohydrate (kcal)	978	340
Carbohydrate (TEI%)	51.4	7.3
Fat (kcal)	639	246
Fat (TEI%)	33.4	6.2
SFA (kcal)	219	93
PUFA (kcal)	149	63
MUFA (kcal)	241	101
Fiber (g)	16	7
Sugar (g)	78	8
Sodium (mg)	1,469	81
Race/Ethnicity		
Hispanic (%)	25.7	−
Non-Hispanic White (%)	46.2	−
Non-Hispanic Black (%)	20.6	−
Other (%)	7.5	−
Family Income to Poverty Ratio	2.5	1.6
Education Level		
Less than high school (%)	10.2	−
High school graduate or GED (%)	76.2	−
Some College or More (%)	13.6	−
Nondrinker (%)	26.5	−
Nonsmoker (%)	50.4	−
Physical Activity (METs)	1,657	2,212
Lipid Profile		
Triglycerides (mg/dL)	138.6	121.2
Total Cholesterol (mg/dL)	197.7	42.7
LDL Cholesterol (mg/dL)	116.6	35.7
HDL Cholesterol (mg/dL)	52.7	16.0
Glycemic Control		
Glucose (mg/dL)	106.9	35.5
Insulin (uU/mL)	13.7	16.3
OGTT (mg/dL)	121.1	53.3
HbA1c (%)	5.7	1.1
Blood Pressure and Adiposity		
Systolic Blood Pressure (mm/Hg)	124.7	19.8
Diastolic Blood Pressure (mm/Hg)	70.3	12.55
Body Fat (%)	32.4	10.7

Participant Characteristics. Body mass index (BMI); Percentage of total energy intake (TEI%); Percentage of total fat intake (FEI%); Standard Deviation (SD)

### Blood lipid profile

**Fig. 1:**
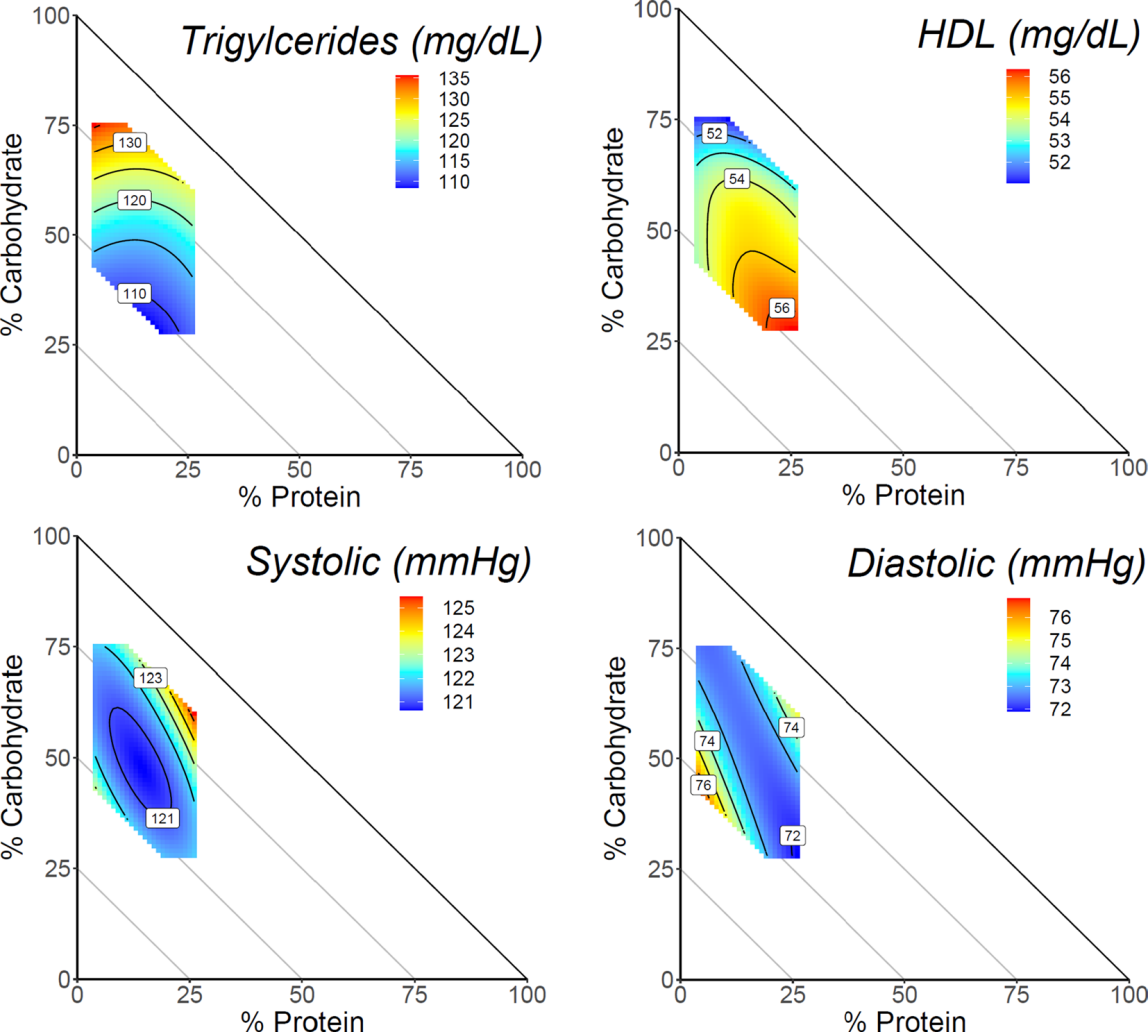
Dietary macronutrient composition and cardiometabolic health markers for females. The mixture triangles show the model predictions of the cardiometabolic health markers with a significant association with macronutrient composition. Predictions were made at mean caloric intake of females (1811 kcal/day) across the sex-specific range of macronutrient percentages in this dataset. Percentage of fat can be inferred as decreasing moving away from the origin, such that each point on the triangle can be summed to equal 100%. Response values are colored such that warm colors display higher values and cooler colors display lower values. Response surfaces were adjusted for age, household income, race/ethnicity, education level, smoking, alcohol, physical activity, BMI, and the Healthy Eating Index

In the fully adjusted model, there was a significant 3-way interaction for macronutrients with triglycerides and HDL cholesterol in females (All *P* ≤ 0.01; Fig. 1; Table 2). Triglycerides were primarily associated with dietary carbohydrate where diets highest in carbohydrate (75%) were associated with the highest fasting triglycerides. Blood HDL values were positively associated with protein and fat where the highest values were associated with diets comprised of lower carbohydrate (30%), moderate protein (25%), and higher fat (45%).

**Table 2:** Associations between Macronutrient Composition and Cardiometabolic Health for Females

Outcome	Model^1^		Model^2^		Model^3^	
	*Dev Exp.*	*P*	*Dev Exp.*	*P*	*Dev Exp.*	*P*
Triglycerides (mg/dL)	6.2%	< 0.01	9.3%	< 0.01	15.2%	< 0.01
Total Cholesterol (mg/dL)	6.7%	0.70	6.9%	0.72	8.0%	0.77
LDL Cholesterol (mg/dL)	4.7%	0.34	4.8%	0.34	6.2%	0.45
HDL Cholesterol (mg/dL)	4.0%	< 0.01	5.2%	< 0.01	14.4%	< 0.01
Glucose (mg/dL)	13.8%	0.20	14.2%	0.20	18.9%	0.24
Insulin (uU/mL)	2.6%	0.28	3.2%	0.39	32.0%	0.47
OGTT (mg/dL)	16.2%	0.02	16.9%	0.03	22.6%	0.07
HbA1c (%)	17.6%	0.60	18.0%	0.58	22.4%	0.64
Systolic Blood Pressure (mm/Hg)	35.4%	< 0.01	35.8%	< 0.01	37.5%	< 0.01
Diastolic Blood Pressure (mm/Hg)	9.4%	< 0.01	9.8%	< 0.01	10.2%	< 0.01
Body Fat (%)	2.6%	0.75	2.6%	0.75	5.0%	0.71

*P*-value reflects the level of significance for macronutrients as a three-dimensional smooth term for triglycerides (*n* = 7,925), total cholesterol (*n* = 15,028), low-density lipoprotein (LDL) cholesterol (*n* = 7,643); high-density lipoprotein (HDL) cholesterol (*n* = 16,100); systolic blood pressure (*n* = 16,355); diastolic blood pressure (*n* = 16,259); body fat percentage (*n* = 1,935); glucose (*n* = 8,026); insulin (*n* = 7,836); oral glucose tolerance test (OGTT; *n* = 3,783); hemoglobin A1C (HbA1c; *n* = 12,077). Percentage of deviance explained (*Dev Exp.*) is shown for the entire model. Body fat percentage was not adjusted for BMI in Model 3.

Model^1^: Adjusted for Age, Household Income

Model^2^: Adjustments as per model 1 + Race/Ethnicity + Education Level

Model^3^: Adjustments as per model 2 + Smoking + Alcohol Intake + Physical Activity + BMI + Healthy Eating Index

**Fig. 2:**
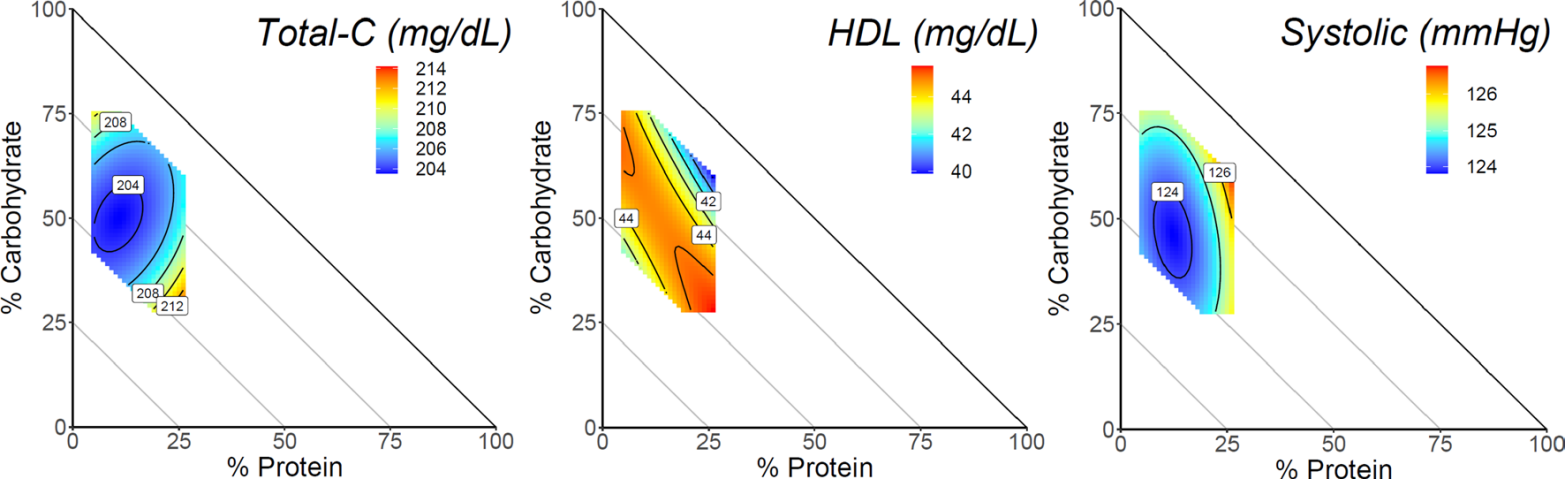
Dietary macronutrient composition and cardiometabolic health markers for males. The mixture triangles show the model predictions of the cardiometabolic health markers with a significant association with macronutrient composition. Predictions were made at mean caloric intake of males (1890 kcal/day) across the sex-specific range of macronutrient percentages in this dataset. The x and y-axis show protein and carbohydrate respectively. Percentage of fat can be inferred as decreasing moving away from the origin, such that each point on the triangle can be summed to equal 100%. Response values are colored such that warm colors display higher values and cooler colors display lower values. Response surfaces were adjusted for age, household income, race/ethnicity, education level, smoking, alcohol, physical activity, BMI, and the Healthy Eating Index

In males, there was a significant 3-way interaction for macronutrients with total cholesterol and HDL cholesterol in the fully adjusted model (All *P* ≤ 0.01; Fig. 2; Table 2). HDL cholesterol was positively associated with dietary fat where the lowest values were observed in diets comprised of lower fat (15%) and moderate protein (25%). Total-C associations were male specific and highest in diets composed the moderate protein (25%), lower carbohydrate (30%), and higher fat (45%). There was no significant association between macronutrient composition and LDL cholesterol in the fully adjusted model for males or females (Table 2).

### Glycemic control

There was no significant association between dietary macronutrient composition and fasting glucose, insulin, or OGTT in the fully adjusted model for either sex (Table 3).

**Table 3:** Associations between Macronutrient Composition and Cardiometabolic Health for Males

Outcome	Model^1^		Model^2^		Model^3^	
	*Dev Exp.*	*P*	*Dev Exp.*	*P*	*Dev Exp.*	*P*
Triglycerides (mg/dL)	3.7%	0.11	5.7%	0.24	11.6%	0.07
Total Cholesterol (mg/dL)	7.3%	< 0.01	8.0%	< 0.01	9.1%	0.01
LDL Cholesterol (mg/dL)	6.2%	0.08	6.8%	0.09	7.8%	0.14
HDL Cholesterol (mg/dL)	1.3%	0.01	2.4%	0.03	14.8%	< 0.01
Glucose (mg/dL)	8.7%	0.12	8.7%	0.12	12.9%	0.12
Insulin (uU/mL)	1.3%	0.66	1.3%	0.66	33.2%	0.18
OGTT (mg/dL)	15.6%	0.68	15.6%	0.68	21.2%	0.75
HbA1c (%)	12.0%	0.57	12.0%	0.57	16.4%	0.50
Systolic Blood Pressure (mm/Hg)	13.8%	0.02	14.2%	0.03	15.3%	0.02
Diastolic Blood Pressure (mm/Hg)	11.5%	0.04	11.6%	0.03	12.5%	0.07
Body Fat (%)	4.8%	0.22	5.1%	0.21	6.0%	0.35

*P*-value reflects the level of significance for macronutrients as a three-dimensional smooth term for triglycerides (*n* = 7,162), total cholesterol (*n* = 13,540), low-density lipoprotein (LDL) cholesterol (*n* = 6,790); high-density lipoprotein (HDL) cholesterol (*n* = 14,613); systolic blood pressure (*n* = 14,962); diastolic blood pressure (*n* = 14,891); body fat percentage (*n* = 1,722); glucose (*n* = 7,215); insulin (*n* = 7,094); oral glucose tolerance test (OGTT; *n* = 3,721); hemoglobin A1C (HbA1c; *n* = 11,322). Percentage of deviance explained (*Dev Exp.*) is shown for the entire model. Body fat percentage was not adjusted for BMI in Model 3.

Model^1^: Adjusted for Age, Household Income

Model^2^: Adjustments as per model 1 + Race/Ethnicity + Education Level

Model^3^: Adjustments as per model 2 + Smoking + Alcohol Intake + Physical Activity + BMI + Healthy Eating Index

### Blood pressure and adiposity

There was a significant 3-way interaction with dietary macronutrients and blood pressure in the fully adjusted model for both males and females (All *P* < 0.01; Fig. 1; Table 2). In females, systolic blood pressure was highest in diets composed of higher protein (30%) coupled with moderate carbohydrate (55%) and lower fat (15%). Diastolic blood pressure was highest across a band of diets with higher fat composition (55%). Lower diastolic blood pressure was observed in a region of higher protein (30%), moderate carbohydrate intake (45%), and moderate fat intake (35%).

A similar association for systolic blood pressure was observed for males while no significant association was detected for diastolic blood pressure (Fig. 2; Table 2). There was no significant association between macronutrient composition with body fat percentage in the fully adjusted model for males or females (Table 3).

### Energy intake

The relationship between dietary macronutrient composition with cardiometabolic health markers across various energy intakes is shown in Supplemental Figs. 2–8. Triglycerides, HDL cholesterol, Total-C, and blood pressure show a similar compositional relationship at varying energy intakes, but with a higher overall effect size at higher total energy intake.

### Healthy eating index components

Response surfaces for the thirteen subcomponents of the HEI are shown in Supplementary Figs. 9–10 and Supplementary Tables 2–3. Response surfaces were similar for both sexes, with higher protein, higher carbohydrate, and lower fat diets achieving the most optimal scores for fruits, vegetables, greens, and beans. Scores for whole grains were most optimal in diets composed of moderate protein, higher carbohydrate, and lower fat. Protein foods and dairy intake scores were positively associated with protein intake while seafood and plant protein scores were highest with a moderate intake of all three macronutrients. Dietary fatty acid ratio was most optimal in diets comprised of lower protein, moderate carbohydrate, and higher fat.

The moderation categories for males and females is shown in Supplementary Figs. 11–12 and Supplementary Tables 2–3. In females and males, diets lowest in carbohydrate and higher in protein had the most optimal sodium score while lower carbohydrate intake was associated with the most optimal refined grain score. There was no significant association between macronutrient composition with added sugars and saturated fat in males or females.

### Additional and sensitivity analyses

In the BMI-stratified analyses for females, the association between macronutrient composition and triglycerides remained only for those with a BMI < 25 (Supplementary Table 4). In overweight or obese females (BMI > 25), significant associations were observed with systolic blood pressure, diastolic blood pressure, and HDL cholesterol. For males with a BMI ≤ 25, the association with macronutrient composition remained only for HDL cholesterol and systolic blood pressure (Supplementary Table 5). In overweight or obese males, there were significant associations with total cholesterol and HDL cholesterol. In the age-stratified analyses, younger females (≤ 48 years) maintained associations between macronutrient composition and triglycerides, systolic blood pressure, and HDL cholesterol (Supplementary Table 6). For older females (> 48 years), the association remained only for triglycerides and systolic blood pressure. Among younger males (≤ 50 years), associations were observed only with HDL cholesterol, while in older males (> 50 years), macronutrient composition was associated with LDL cholesterol, total cholesterol, and systolic blood pressure (Supplementary Table 7)

When we excluded participants with comorbid conditions or taking related medication, only associations for macronutrients with HDL cholesterol and systolic blood pressure remained significant in females while HDL cholesterol and diastolic blood pressure remained significant in males (Supplementary Tables 8–9). All associations remained the same when excluding pregnant women from the analysis (Supplementary Table 10).When we excluded participants who had a single 24-hour dietary recall, only diastolic blood pressure had a significant association with macronutrient composition (Supplementary Tables 11–12). 

## Discussion

Cardiometabolic health is widely recognized as a fundamental mediator between the associations established with nutrition, NCDs, and mortality [38, 39]. Dietary macronutrients serve as integral components in the dynamic relationship between diet and cardiometabolic health. In the fully adjusted model of our analysis, we revealed cardiometabolic health significantly differed across dietary macronutrient profiles regardless of dietary quality. Specifically, there was a complex nonlinear relationship between macronutrient composition with unique sex-specific differences in associations for triglycerides, total-cholesterol, HDL cholesterol, and blood pressure.

The current findings revealed several important trade-offs in the relationship between macronutrient composition and blood lipid profile. Specifically, diets with higher overall carbohydrate content were associated with higher fasting triglycerides in females. This is in direct contrast to HDL where in both sexes the highest values were observed in diets containing more dietary fat. Associations with Total-C were unique to men where higher protein coupled with higher fat was associated with the greatest Total-C. This trade-off for dietary macronutrients with blood lipids aligns with a recent meta-analysis of 32 randomized control trials assessing the impact of long-term (> 12 months) low fat and high fat diets on blood lipid profile [40]. Specifically, that high fat diets appear positively associated with HDL and negatively associated with triglycerides while no associations were observed for LDL. Similar findings were revealed in the Prospective Urban Rural Epidemiology (PURE) study which analyzed associations of dietary data with cardiometabolic health across 18 countries (*n* = 125,287). This study showed dietary fat was similarly associated with HDL cholesterol and triglycerides while the link between dietary fat and LDL cholesterol was more strongly associated with fat quality rather than total fat intake [41].

Poor glycemic control has commonly been associated with the intake of carbohydrates, particularly refined carbohydrates with a higher glycemic load [42]. Research examining the relationship between dietary fat and glycemic control has often directly compared isocaloric replacement with carbohydrate without exploring differences across protein intake. Higher protein diets have been shown to stimulate insulin secretion, which in the long term has been associated with an increased risk of type II diabetes [43]. In the present study, we revealed no significant associations of macronutrient composition with markers of glycemic control. These findings point toward the possibility that other factors such as physical activity, BMI, and dietary quality (e.g., fruits, vegetables, refined grains, and fatty acid profile) may play a more influential role in glycemic control [44–46].

Previous research has often explored the impact of dietary macronutrient composition on weight loss in long-term intervention trials which suggest that higher fat coupled with higher protein is beneficial [19]. This effect has been thought to be partially due to the protein leverage hypothesis, where experimental and observational evidence supports that protein appetite in combination with low dietary protein drives excess energy intake [47, 48]. Therefore, sufficient dietary protein intake could reduce excess energy consumption supporting weight loss and ultimately improving overall body composition. In contrast, the present study found no association between macronutrient composition and body fat percentage. However, much of the research exploring the role of macronutrients on body composition is examined in a hypocaloric environment [19] which may explain differences observed in the context of long-term *ad libitum* diet composition. Lastly, the current findings also revealed a relationship between macronutrient and blood pressure. Higher overall systolic blood pressure was observed for those consuming diets higher in protein and lower in fat. In females, diastolic blood pressure was highest in a band of diet compositions at the upper range of dietary fat intake (55%). Limited evidence exists for the direct relationship between macronutrients and blood pressure. However, the total effect observed in this study was limited and may stem from underlying dietary components not fully captured in this analysis.

This study also highlights distinct sex-specific differences in the associations between dietary macronutrient composition and cardiometabolic health. Notably, associations with triglycerides were specific to females with a strong positive association with carbohydrate intake. For males, we observed a unique association for Total-C where diets with the upper range of reported protein intake and higher contribution of energy from dietary fat had the highest Total-C levels. These findings may be partly related to sex-specific influencers of metabolism such as hormones, skeletal muscle mass, and body fat distribution [49]. For example, estrogen plays an important role in chylomicron metabolism and reverse cholesterol transport which may explain some of the differences in blood lipid profile [50, 51].

An increasing emphasis has been made on improving diet quality by focusing on specific foods and dietary patterns [52, 53]. However, the present study revealed that there was a significant association of several cardiometabolic health markers across the spectrum of macronutrient composition. Although the current US dietary guidelines do not provide explicit macronutrient ranges, the Institute of Medicine (IOM) has outlined a set of acceptable macronutrient ranges consisting of 10–35% protein, 45–65% carbohydrate, and 20–35% fat [54]. These ranges are designed to reduce the risk of noncommunicable diseases while taking into consideration the ability to meet micronutrient requirements. Our study reveals that even after adjusting for diet quality, several cardiometabolic health markers span across both the highest and lowest ends of these recommended ranges. These findings underscore the need for more detailed macronutrient recommendations and to further evaluate how these requirements might vary across different stages of life.

### Strengths and limitations

Nutrition research frequently adopts a one-variable-at-a-time approach, often missing the intricate interplay between various nutrients. A key strength of this study is that we have taken a multidimensional approach to analyze the associations between nutrition and cardiometabolic health. Notably, these results remained the same after adjustment for the HEI which acts as a measure of dietary quality by quantifying the level of adherence to the US dietary guidelines. Although we have adjusted for dietary quality using the HEI, this does not fully capture all aspects of diet quality and does not account for individual variation in nutrient needs by age or sex [55]. To further explore how other components of diet quality may shape these findings, we examined how the specific food components of the HEI were associated with macronutrient composition. We show that nearly all food components in the HEI differed significantly in relation to macronutrient composition. However, this study did not examine aspects of individual macronutrient quality, such as distinguishing plant vs. animal macronutrients and level of processing which have distinctly different health effects on cardiometabolic health [56, 57]. Therefore, these results should be viewed as part of a broader continuum of nutritional research that considers the interplay between macronutrients, specific foods, and overall dietary patterns.

Despite the novel multidimensional approach undertaken, many of the observations in the study may be partially influenced by inter-individual variability that exists in how individuals respond to dietary components and diet composition [58]. Particularly, aspects such as genetics [13] and gut microbial health [32, 59] play an integral role in how diet influences cardiometabolic health and due to the nature of this study design could not be thoroughly investigated. Other limitations of these findings include the inherent risks observed in nutritional epidemiology such as the risk of over and underreporting dietary intake. Moreover, this study included a summary of cross-sectional nutritional data which precludes assuming causality between the observed relationships.

## Conclusion

The GFN offers a unique perspective on the dynamic relationship between nutrition and human health. Our study identified a complex sex-specific relationship between macronutrient composition and several markers of cardiometabolic health. To gain a more comprehensive understanding of these associations, future research is needed to investigate how they vary across different dietary patterns and age groups.

## Electronic supplementary material

Below is the link to the electronic supplementary material.

Supplementary Material 1

## Data Availability

Data from the National Health and Nutrition Examination Survey is publicly available online (https://www.cdc.gov/nchs/nhanes/index.htm).
