# Supplementary material for: Associations between dietary macronutrient composition and cardiometabolic health: data from NHANES 1999–2014
Source: Eur J Nutr. 2024 Dec 7;64(1):41. doi: 10.1007/s00394-024-03523-7 (PMC11624254; doi:10.1007/s00394-024-03523-7)
Supplement: Supplementary file 1 — Supplementary Material 1 [file 394_2024_3523_MOESM1_ESM.docx]

**ONLINE SUPPLEMENTARY MATERIAL**

**Associations Between Dietary Macronutrient Composition and Cardiometabolic Health: Data from NHANES 1999-2014**

Nicholas A. Koemel^1,2^, Alistair M. Senior^1,3^, Nasser Laouali^4,5,6^, David S. Celermajer^2^, Amanda Grech^1,3^, Helen M. Parker^1,2^, Stephen J. Simpson^1,3^, David Raubenheimer^1,3^, Timothy P. Gill^1,2,7*^, Michael R. Skilton^2^

^1^Charles Perkins Centre, The University of Sydney, Sydney, Australia

^2^Sydney Medical School, The University of Sydney, Sydney, Australia

^3^School of Life and Environmental Sciences, The University of Sydney, Sydney, Australia

^4^Université Paris-Saclay, CESP UMR1018, UVSQ, Inserm, Gustave Roussy, Villejuif, Paris, France

^5^Department of Biostatistics and Epidemiology, School of Public Health and Health Sciences, University of Massachusetts, Amherst, Massachusetts, USA

^6^Scripps Institution of Oceanography, University of California, San Diego, US

^7^Susan Wakil School of Nursing and Midwifery, The University of Sydney, Sydney, Australia

**TABLE OF CONTENTS**

Page

I. **SUPPLEMENTARY FIGURES**

Participant Flowchart 2

Associations between Macronutrient Composition and Triglycerides Across Energy Intakes in Females 3

Associations between Macronutrient Composition and HDL Cholesterol Across Energy Intakes in Females 4

Associations between Macronutrient Composition and Systolic Blood Pressure Across Energy Intakes in Females 5

Associations between Macronutrient Composition and Diastolic Blood Pressure Across Energy Intakes in Females 6

Associations between Macronutrient Composition and Total Cholesterol Across Energy Intakes in Males 7

Associations between Macronutrient Composition and HDL Cholesterol Across Energy Intakes in Males 8

Associations between Macronutrient Composition and Systolic Blood Pressure Across Energy Intakes in Males 9

Dietary Macronutrient Composition and Adequacy Components of the Healthy Eating Index in Females 10

Dietary Macronutrient Composition and Adequacy Components of the Healthy Eating Index in Males 11

Dietary Macronutrient Composition and Moderation Components of the Healthy Eating Index Females 12

Dietary Macronutrient Composition and Moderation Components of the Healthy Eating Index in Males 13

II. **SUPPLEMENTARY TABLES**

Healthy Eating Index 2015 Components and Scoring 14

Associations between Macronutrient Composition with Components of the Healthy Eating Index in Females 15

Associations Between Macronutrient Composition with Components of the Healthy Eating Index in Males 16

Associations Between Macronutrient Composition and Cardiometabolic Health: Stratified by BMI in Females 17

Associations Between Macronutrient Composition and Cardiometabolic Health: Stratified by BMI in Males 19

Associations Between Macronutrient Composition and Cardiometabolic Health: Stratified by Age in Females 21

Associations Between Macronutrient Composition and Cardiometabolic Health: Stratified by Age in Males 23

Associations between Macronutrient Composition and Cardiometabolic Health: Cardiometabolic Sensitivity in Females 25

Associations between Macronutrient Composition and Cardiometabolic Health: Cardiometabolic Sensitivity in Males 26

Associations between Macronutrient Composition and Cardiometabolic Health: Pregnancy Sensitivity 27

Associations between Macronutrient Composition and Cardiometabolic Health: Dietary Recall Sensitivity Including Only Individuals with Two Completed 24-hour Recalls in Females 28

Associations between Macronutrient Composition and Cardiometabolic Health: Dietary Recall Sensitivity Including Only Individuals with Two Completed 24-hour Recalls in Males 29

**
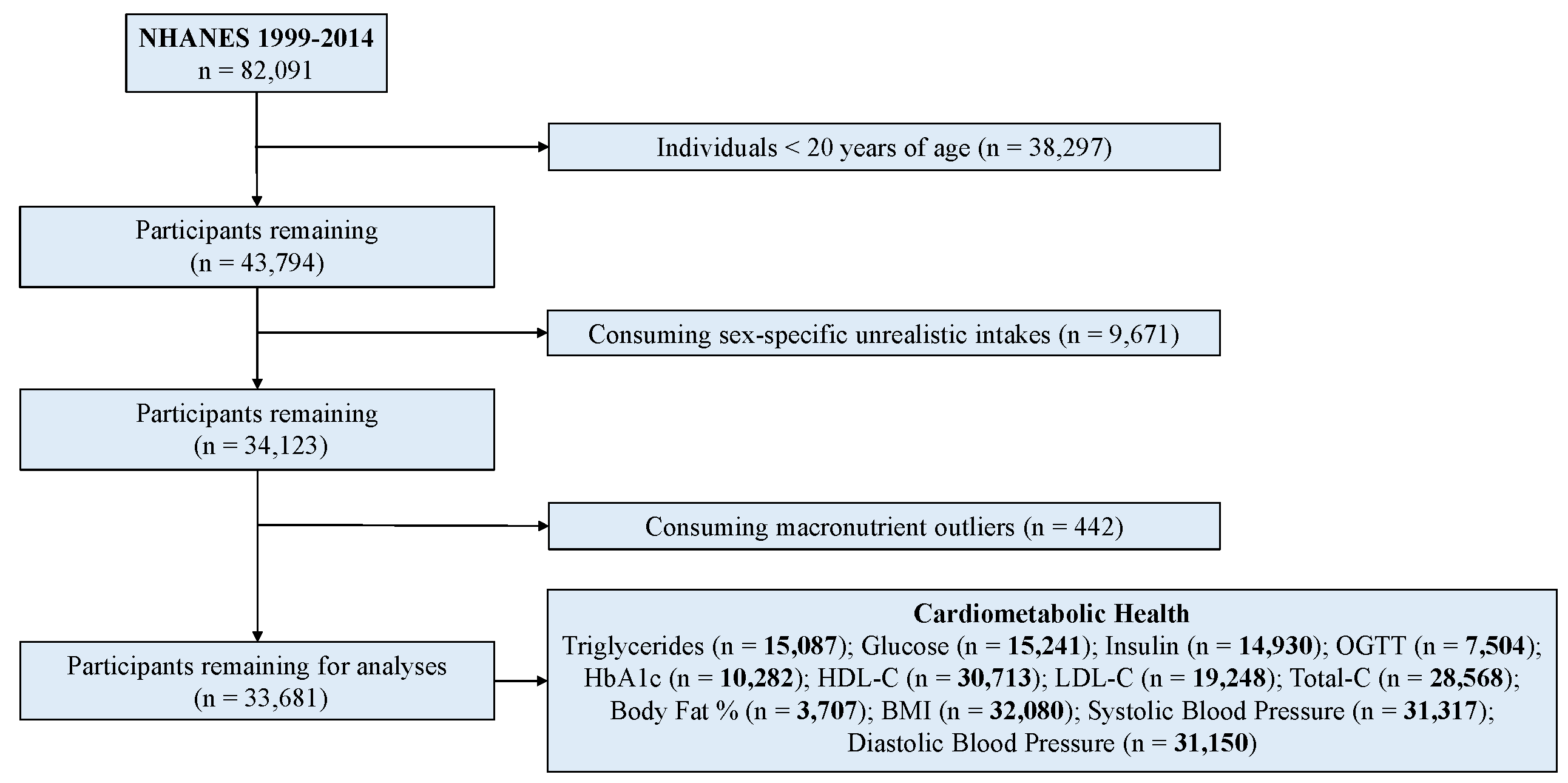
**

**Supplemental Figure 1. Participant Flow Chart.**

**
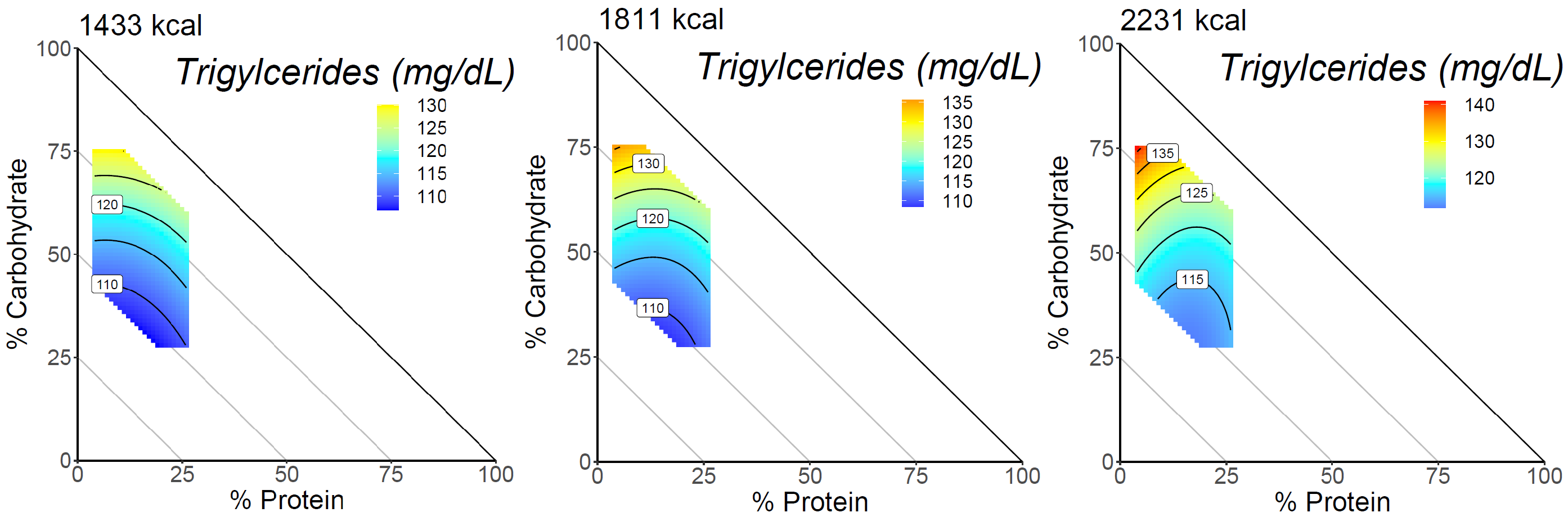
**

**Supplementary Figure 2. Associations between Macronutrient Composition and Triglycerides Across Energy Intakes in Females.** The mixture triangles show the model predictions of the fasting triglycerides at the 25^th^, 50^th^, and 75^th^ percentile of total energy intake from left to right. Predictions were made for the range of macronutrient percentages in this dataset. The x and y-axis show protein and carbohydrate respectively. Percentage of fat can be inferred as decreasing moving away from the origin, such that each point on the triangle can be summed to equal 100%. Response values are colored such that warm colors display higher values and cooler colors display lower values. Response surfaces were adjusted for age, household income, race/ethnicity, education level, smoking, alcohol, physical activity, BMI, and the Healthy Eating Index.

**
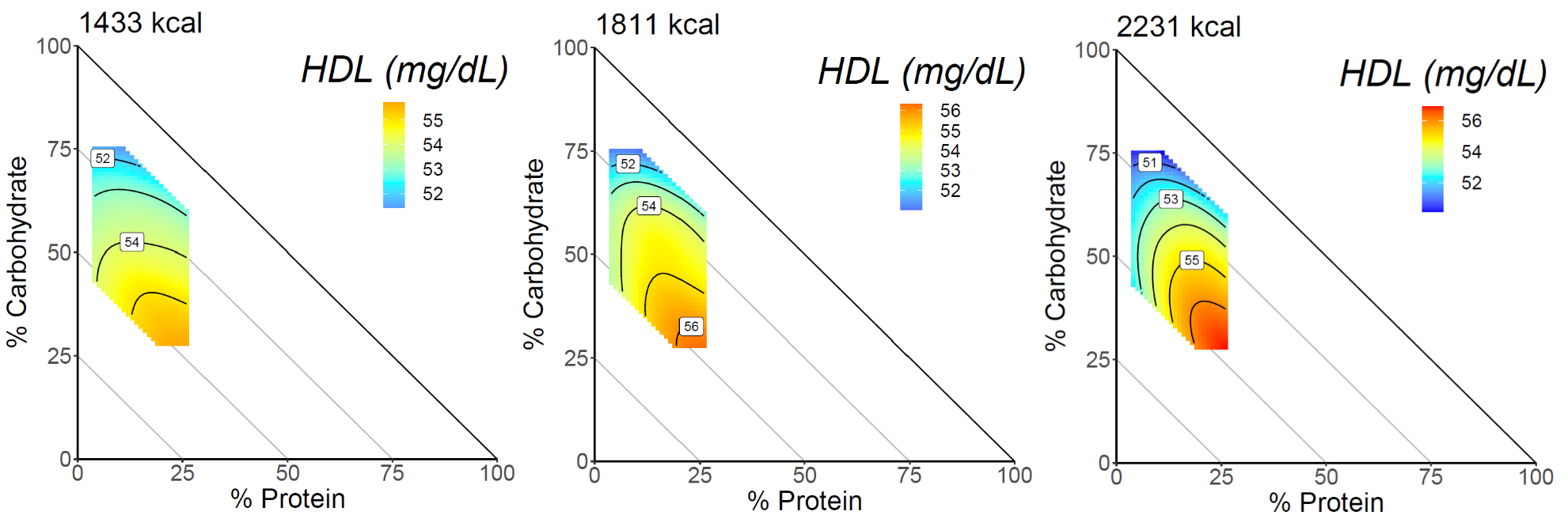
**

**Supplementary Figure 3.** **Associations between Macronutrient Composition and HDL Cholesterol Across Energy Intakes in Females.** The mixture triangles show the model predictions of the HDL cholesterol at the 25^th^, 50^th^, and 75^th^ percentile of total energy intake from left to right. Predictions were made for the range of macronutrient percentages in this dataset. The x and y-axis show protein and carbohydrate respectively. Percentage of fat can be inferred as decreasing moving away from the origin, such that each point on the triangle can be summed to equal 100%. Response values are colored such that warm colors display higher values and cooler colors display lower values. Response surfaces were adjusted for age, household income, race/ethnicity, education level, smoking, alcohol, physical activity, BMI, and the Healthy Eating Index.

**
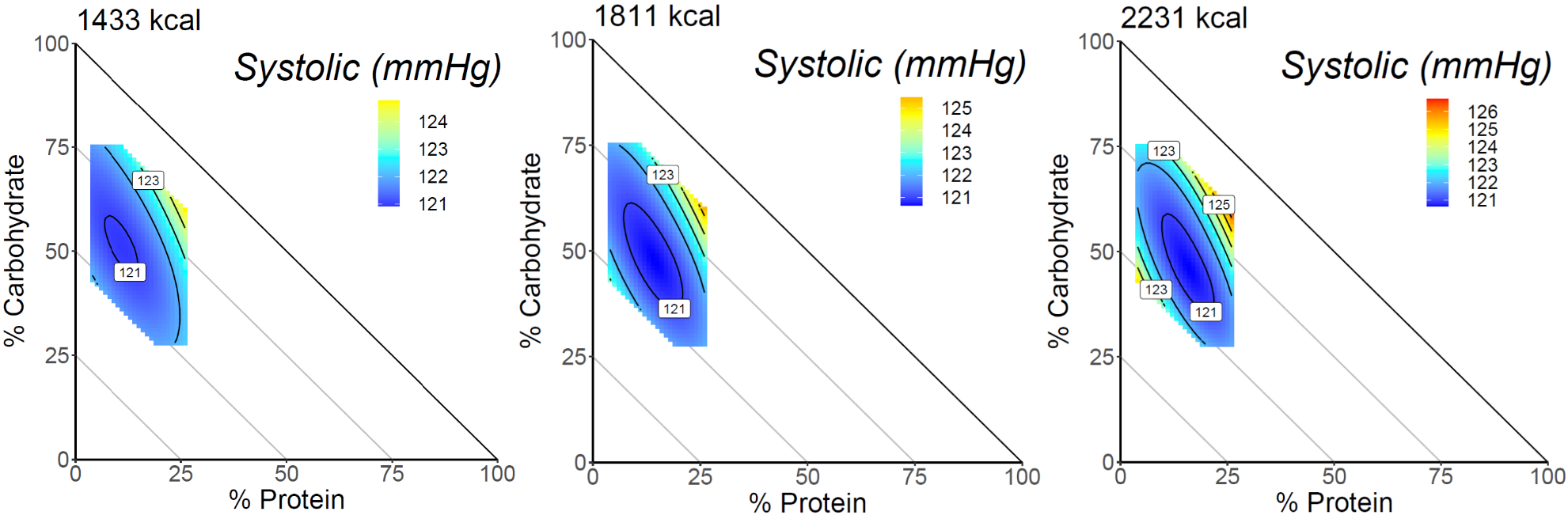
**

**Supplementary Figure 4. Associations between Macronutrient Composition and Systolic Blood Pressure Across Energy Intakes in Females.** The mixture triangles show the model predictions of the systolic blood pressure at the 25^th^, 50^th^, and 75^th^ percentile of total energy intake from left to right. Predictions were made for the range of macronutrient percentages in this dataset. The x and y-axis show protein and carbohydrate respectively. Percentage of fat can be inferred as decreasing moving away from the origin, such that each point on the triangle can be summed to equal 100%. Response values are colored such that warm colors display higher values and cooler colors display lower values. Response surfaces were adjusted for age, household income, race/ethnicity, education level, smoking, alcohol, physical activity, BMI, and the Healthy Eating Index.

**
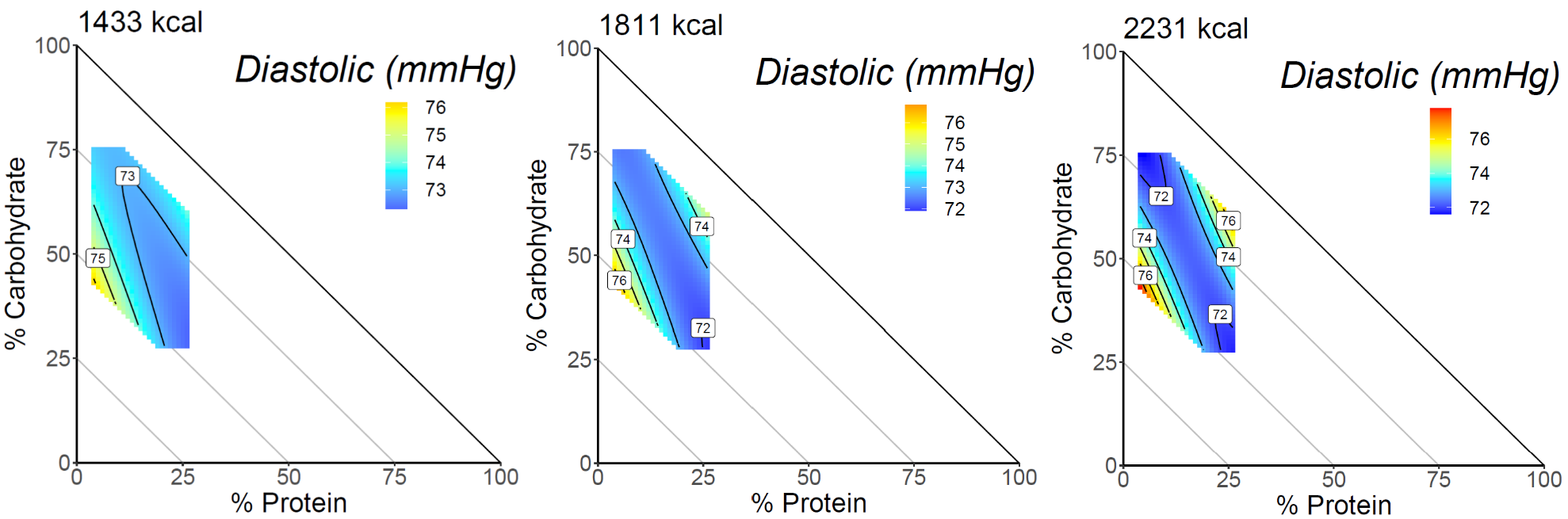
**

**Supplementary Figure 5. Associations between Macronutrient Composition and Diastolic Blood Pressure Across Energy Intakes in Females.** The mixture triangles show the model predictions of the diastolic blood pressure at the 25^th^, 50^th^, and 75^th^ percentile of total energy intake from left to right. Predictions were made for the range of macronutrient percentages in this dataset. The x and y-axis show protein and carbohydrate respectively. Percentage of fat can be inferred as decreasing moving away from the origin, such that each point on the triangle can be summed to equal 100%. Response values are colored such that warm colors display higher values and cooler colors display lower values. Response surfaces were adjusted for age, household income, race/ethnicity, education level, smoking, alcohol, physical activity, BMI, and the Healthy Eating Index.

**
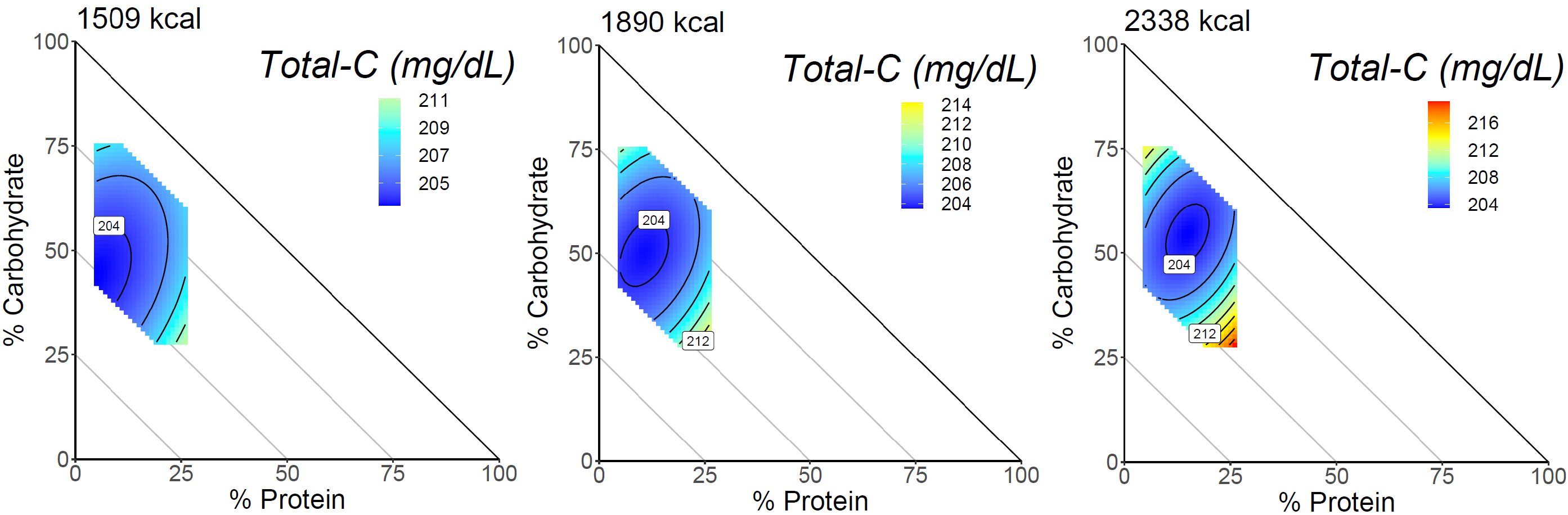
**

**Supplementary Figure 6. Associations between Macronutrient Composition and Total Cholesterol Across Energy Intakes in Males.** The mixture triangles show the model predictions of the total cholesterol at the 25^th^, 50^th^, and 75^th^ percentile of total energy intake from left to right. Predictions were made for the range of macronutrient percentages in this dataset. The x and y-axis show protein and carbohydrate respectively. Percentage of fat can be inferred as decreasing moving away from the origin, such that each point on the triangle can be summed to equal 100%. Response values are colored such that warm colors display higher values and cooler colors display lower values. Response surfaces were adjusted for age, household income, race/ethnicity, education level, smoking, alcohol, physical activity, BMI, and the Healthy Eating Index.

**
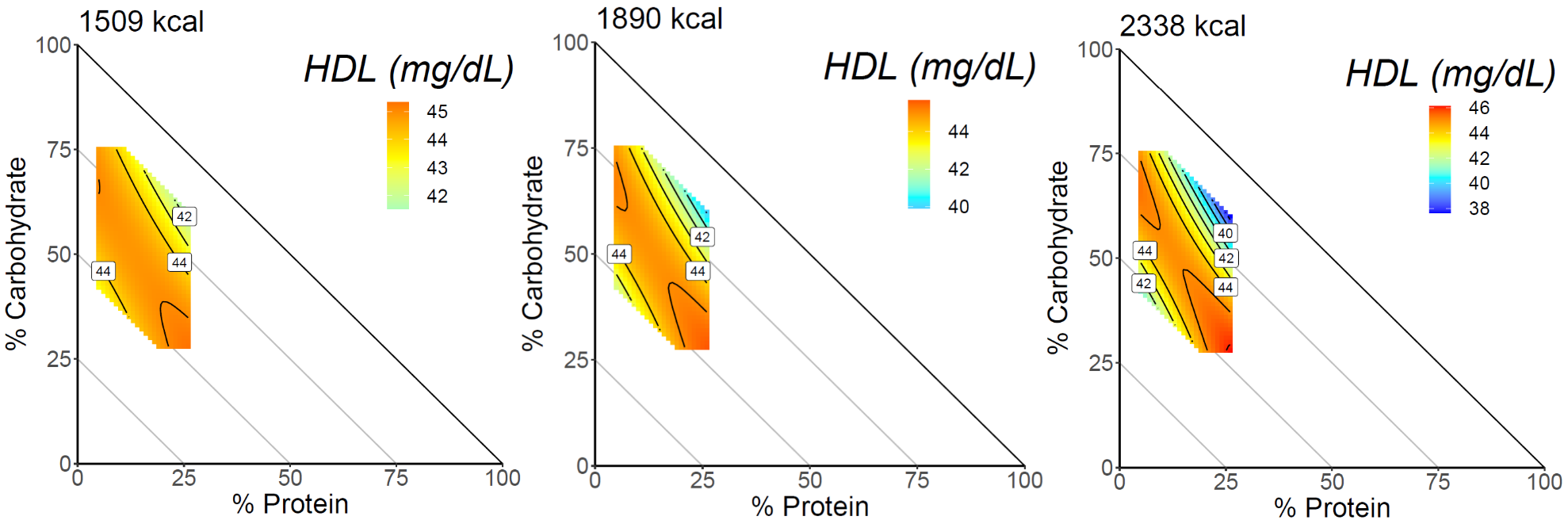
**

**Supplementary Figure 7. Associations between Macronutrient Composition and HDL Cholesterol Across Energy Intakes in Males.** The mixture triangles show the model predictions of the HDL cholesterol at the 25^th^, 50^th^, and 75^th^ percentile of total energy intake from left to right. Predictions were made for the range of macronutrient percentages in this dataset. The x and y-axis show protein and carbohydrate respectively. Percentage of fat can be inferred as decreasing moving away from the origin, such that each point on the triangle can be summed to equal 100%. Response values are colored such that warm colors display higher values and cooler colors display lower values. Response surfaces were adjusted for age, household income, race/ethnicity, education level, smoking, alcohol, physical activity, BMI, and the Healthy Eating Index.

**
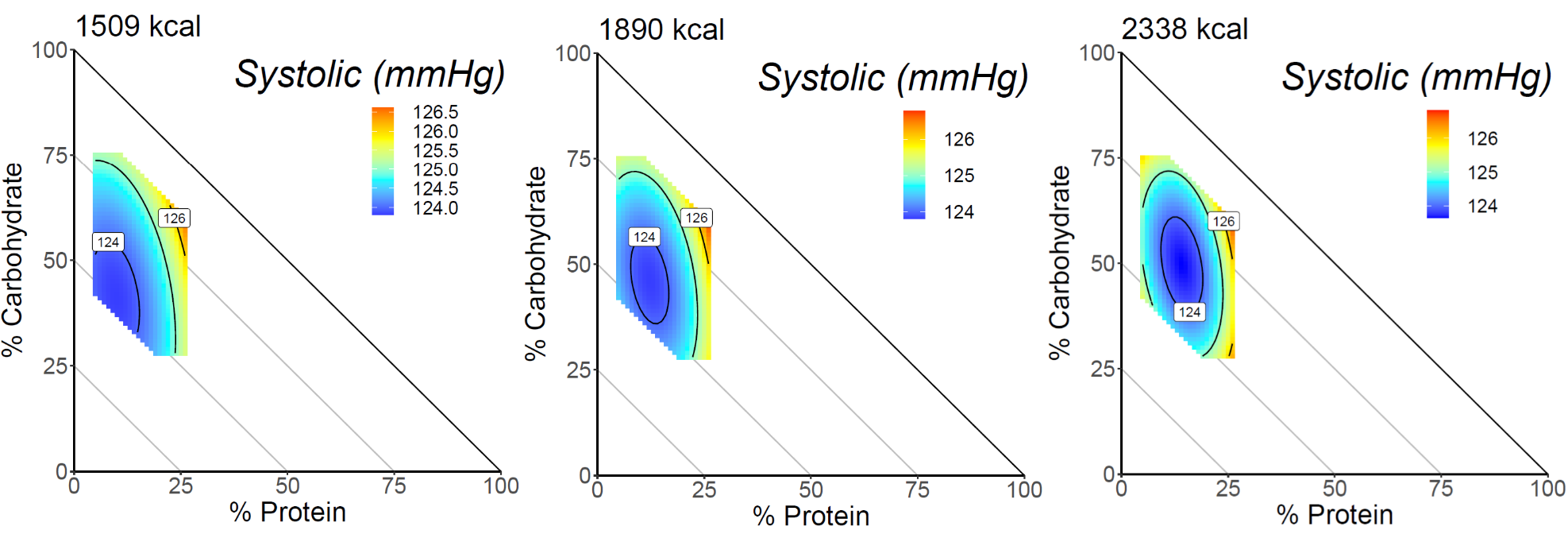
**

**Supplementary Figure 8. Associations between Macronutrient Composition and Systolic Blood Pressure Across Energy Intakes in Males.** The mixture triangles show the model predictions of the systolic blood pressure at the 25^th^, 50^th^, and 75^th^ percentile of total energy intake from left to right. Predictions were made for the range of macronutrient percentages in this dataset. The x and y-axis show protein and carbohydrate respectively. Percentage of fat can be inferred as decreasing moving away from the origin, such that each point on the triangle can be summed to equal 100%. Response values are colored such that warm colors display higher values and cooler colors display lower values. Response surfaces were adjusted for age, household income, race/ethnicity, education level, smoking, alcohol, physical activity, BMI, and the Healthy Eating Index.


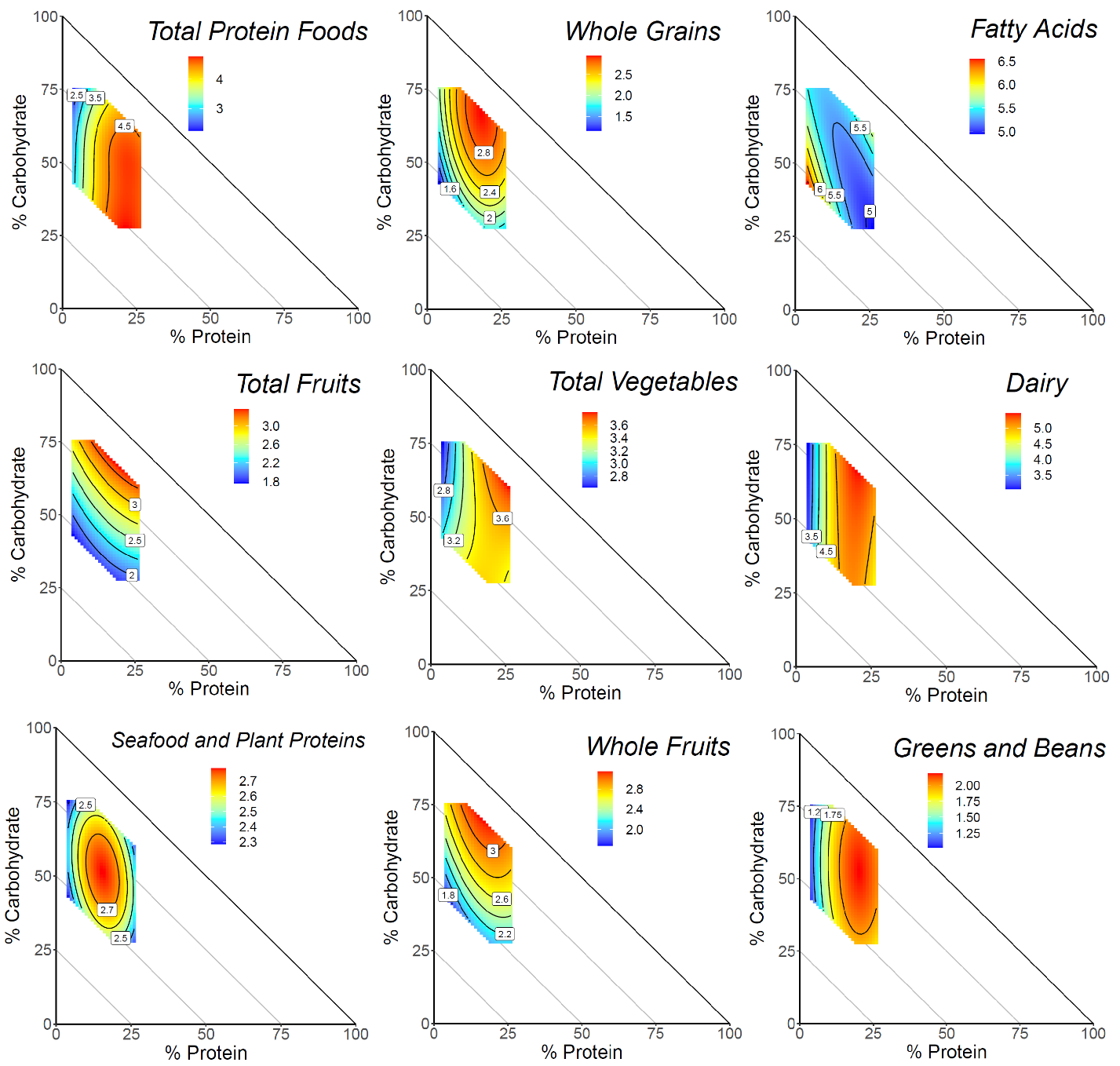


**Supplementary Figure 9. Dietary Macronutrient Composition and Adequacy Components of the Healthy Eating Index in Females.** The mixture triangles show the model predictions of the adequacy components of the Healthy Eating Index with a significant association with macronutrient composition. Predictions were made at mean caloric intake of females (1811 kcal/day) across the sex-specific range of macronutrient percentages in this dataset. The x and y-axis show protein and carbohydrate respectively. Percentage of fat can be inferred as decreasing moving away from the origin, such that each point on the triangle can be summed to equal 100%. Response values are colored such that warm colors display higher values and cooler colors display lower values. Response surfaces were adjusted for age, household income, race/ethnicity, education level, smoking, alcohol, physical activity, BMI, and the Healthy Eating Index.


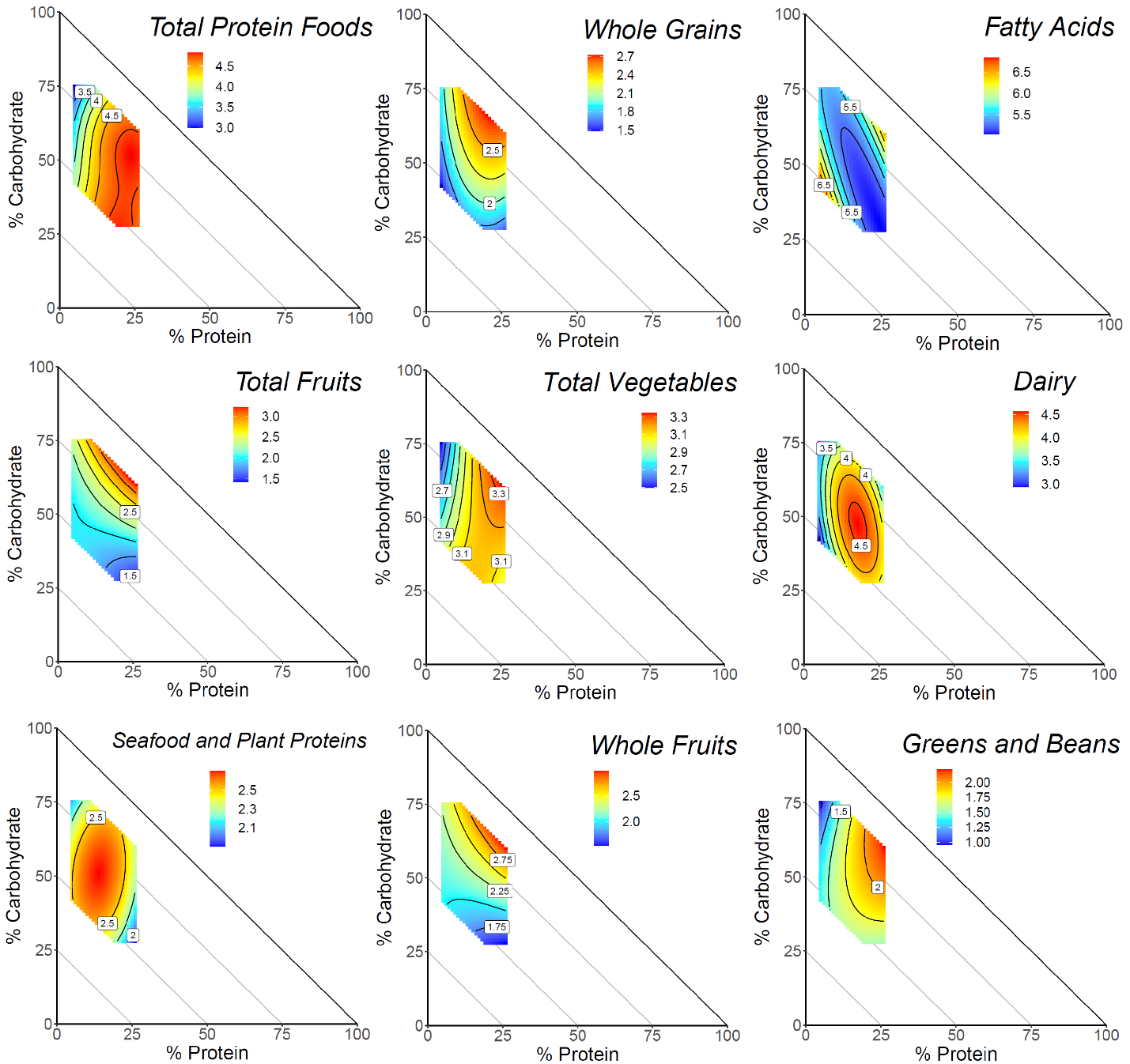


**Supplementary Figure 10. Dietary Macronutrient Composition and Adequacy Components of the Healthy Eating Index in Males.** The mixture triangles show the model predictions of the adequacy components of the Healthy Eating Index with a significant association with macronutrient composition. Predictions were made at mean caloric intake of males (1890 kcal/day) across the sex-specific range of macronutrient percentages in this dataset. The x and y-axis show protein and carbohydrate respectively. Percentage of fat can be inferred as decreasing moving away from the origin, such that each point on the triangle can be summed to equal 100%. Response values are colored such that warm colors display higher values and cooler colors display lower values. Response surfaces were adjusted for age, household income, race/ethnicity, education level, smoking, alcohol, physical activity, BMI, and the Healthy Eating Index.

**
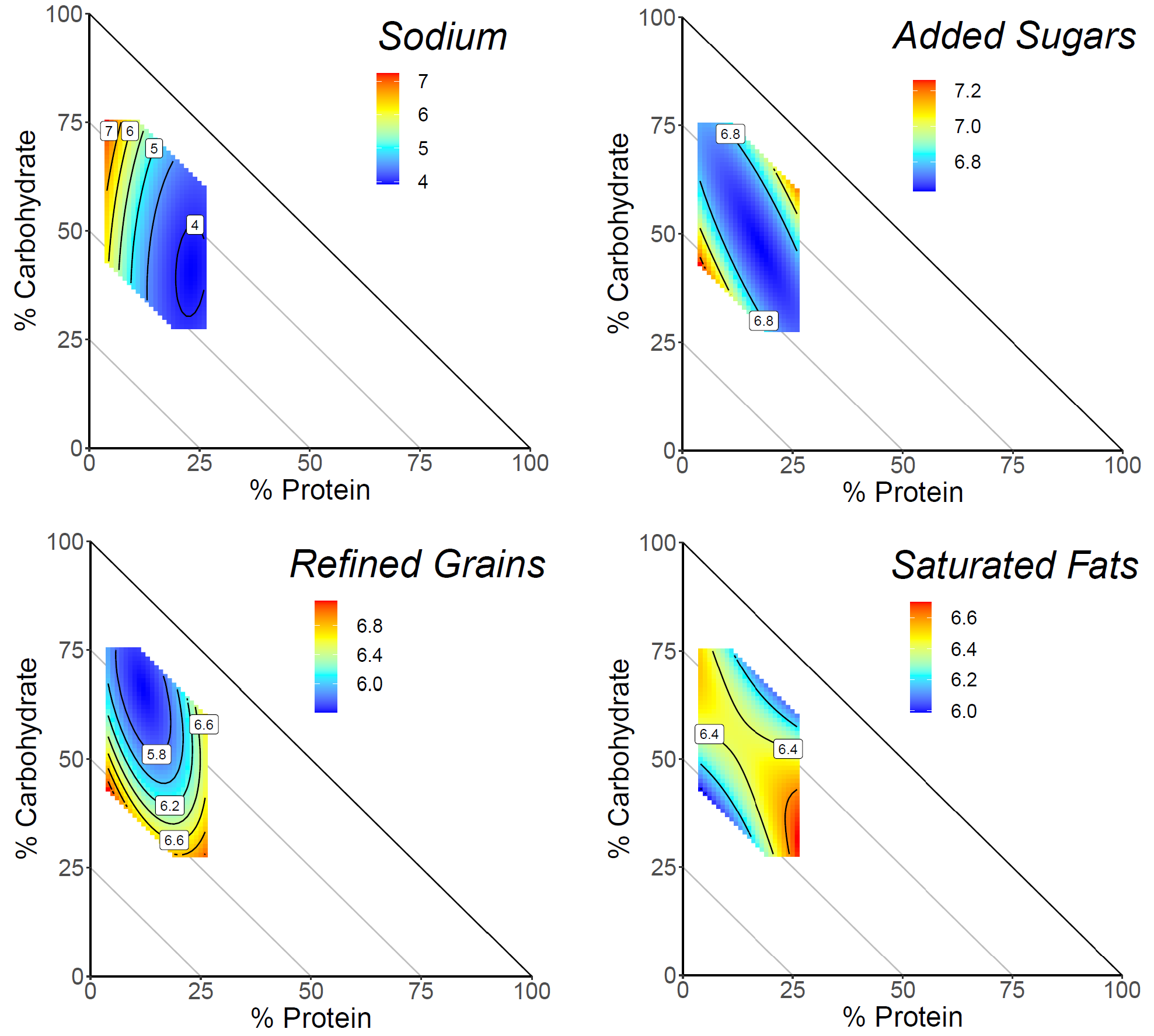
**

**Supplementary Figure 11. Dietary Macronutrient Composition and Moderation Components of the Healthy Eating Index Females.** The mixture triangles show the model predictions of the moderation components of the Healthy Eating Index with a significant association with macronutrient composition. Predictions were made at mean caloric intake of females (1811 kcal/day) across the sex-specific range of macronutrient percentages in this dataset. The x and y-axis show protein and carbohydrate respectively. Percentage of fat can be inferred as decreasing moving away from the origin, such that each point on the triangle can be summed to equal 100%. Response values are colored such that warm colors display higher values and cooler colors display lower values. Response surfaces were adjusted for age, household income, race/ethnicity, education level, smoking, alcohol, physical activity, BMI, and the Healthy Eating Index.

**
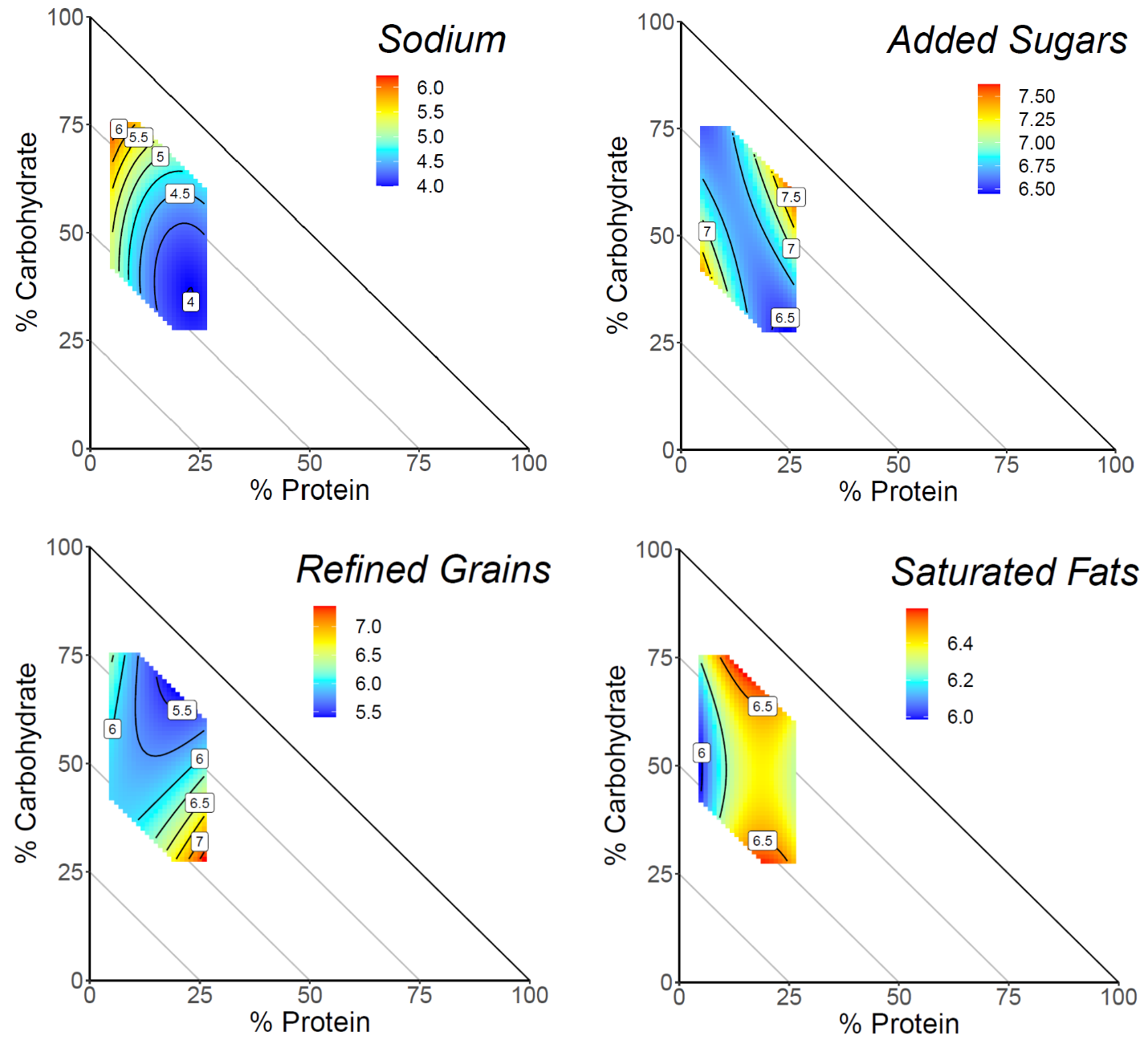
**

**Supplementary Figure 12. Dietary Macronutrient Composition and Moderation Components of the Healthy Eating Index in Males.** The mixture triangles show the model predictions of the moderation components of the Healthy Eating Index with a significant association with macronutrient composition. Predictions were made at mean caloric intake of males (1890 kcal/day) across the sex-specific range of macronutrient percentages in this dataset. The x and y-axis show protein and carbohydrate respectively. Percentage of fat can be inferred as decreasing moving away from the origin, such that each point on the triangle can be summed to equal 100%. Response values are colored such that warm colors display higher values and cooler colors display lower values. Response surfaces were adjusted for age, household income, race/ethnicity, education level, smoking, alcohol, physical activity, BMI, and the Healthy Eating Index.

**Supplementary Table 1**. Healthy Eating Index 2015 Components and Scoring

| **Component** | **Maximum Points** | **Standard for maximum score** | **Standard for minimum score of zero** |
| --- | --- | --- | --- |
| **Adequacy** | | | |
| Total Fruits[^2^](https://epi.grants.cancer.gov/hei/developing.html#f2b) | 5 | ≥0.8 cup equiv. per 1,000 kcal | No Fruits |
| Whole Fruits[^3^](https://epi.grants.cancer.gov/hei/developing.html#f3b) | 5 | ≥0.4 cup equiv. per 1,000 kcal | No Whole Fruits |
| Total Vegetables[^4^](https://epi.grants.cancer.gov/hei/developing.html#f4b) | 5 | ≥1.1 cup equiv. per 1,000 kcal | No Vegetables |
| Greens and Beans[^4^](https://epi.grants.cancer.gov/hei/developing.html#f4b) | 5 | ≥0.2 cup equiv. per 1,000 kcal | No Dark Green Vegetables or Legumes |
| Whole Grains | 10 | ≥1.5 oz equiv. per 1,000 kcal | No Whole Grains |
| Dairy[^5^](https://epi.grants.cancer.gov/hei/developing.html#f5b) | 10 | ≥1.3 cup equiv. per 1,000 kcal | No Dairy |
| Total Protein Foods[^6^](https://epi.grants.cancer.gov/hei/developing.html#f6b) | 5 | ≥2.5 oz equiv. per 1,000 kcal | No Protein Foods |
| Seafood and Plant Proteins[^6^](https://epi.grants.cancer.gov/hei/developing.html#f6b)^,^[^7^](https://epi.grants.cancer.gov/hei/developing.html#f7b) | 5 | ≥0.8 oz equiv. per 1,000 kcal | No Seafood or Plant Proteins |
| Fatty Acids[^7^](https://epi.grants.cancer.gov/hei/developing.html#f7b) | 10 | (PUFAs + MUFAs)/SFAs ≥2.5 | (PUFAs + MUFAs)/SFAs ≤1.2 |
| **Moderation** | | | |
| Refined Grains | 10 | ≤1.8 oz equiv. per 1,000 kcal | ≥4.3 oz equiv. per 1,000 kcal |
| Sodium | 10 | ≤1.1 gram per 1,000 kcal | ≥2.0 grams per 1,000 kcal |
| Added Sugars | 10 | ≤6.5% of energy | ≥26% of energy |
| Saturated Fats | 10 | ≤8% of energy | ≥16% of energy |

**1:** The HEI-2020 components and scoring standards are the same as the HEI-2015. Intakes between the minimum and maximum standards are scored proportionately. The total HEI score is the sum of the adequacy components (i.e. foods to eat more of for good health) and moderation components (i.e. foods to limit for good health).

**2:** Includes 100% fruit juice.

**3:** Includes all forms except juice.

**4:** Includes legumes (beans and peas).

**5:** Includes all milk products, such as fluid milk, yogurt, and cheese, and fortified soy beverages.

**6:** Includes seafood, nuts, seeds, soy products (other than beverages), and beans, peas, and lentils.

**7:** Ratio of poly- and monounsaturated fatty acids (PUFAs and MUFAs) to saturated fatty acids (SFAs).

For the years 1999-2002, a moderation category known as “empty calories” was used which includes a score for the percentage of total energy intake from solid fats, added sugars, and alcohol^14^.

| **Supplemental Table 2.** Associations Between Macronutrient Composition with Components of the Healthy Eating Index in Females | | |
| --- | --- | --- |
| **Outcome** | **Model^1^** | |
| **Adequacy** | *Dev Exp.* | *P* |
| Total Fruits | 8.7% | <0.01 |
| Whole Fruits | 10.2% | <0.01 |
| Total Vegetables | 4.5% | <0.01 |
| Greens and Beans | 2.9% | <0.01 |
| Whole Grains | 7.2% | <0.01 |
| Dairy | 3.7% | <0.01 |
| Total Protein Foods | 8.4% | <0.01 |
| Seafood and Plant Proteins | 3.1% | <0.01 |
| Fatty Acids | 1.6% | 0.01 |
| **Moderation** | ─ | ─ |
| Refined Grains | 5.0% | <0.01 |
| Sodium | 3.0% | <0.01 |
| Added Sugars | 5.6% | 0.98 |
| Saturated Fats | 1.5% | 0.88 |

^1^*P*-value reflects the level of significance for macronutrients as a three-dimensional smooth term for the adequacy and moderation components of the Healthy Eating Index. Percentage of deviance explained (*Dev Exp.*) is shown for the entire model. Models were adjusted for age, household income, race/ethnicity, education level, smoking, alcohol, physical activity, BMI, and the Healthy Eating Index.

| **Supplemental Table 3.** Associations Between Macronutrient Composition with Components of the Healthy Eating Index in Males | | |
| --- | --- | --- |
| **Outcome** | **Model^1^** | |
| **Adequacy** | *Dev Exp.* | *P* |
| Total Fruits | 8.6% | <0.01 |
| Whole Fruits | 9.0% | <0.01 |
| Total Vegetables | 3.9% | <0.01 |
| Greens and Beans | 2.4% | <0.01 |
| Whole Grains | 8.3% | <0.01 |
| Dairy | 3.2% | <0.01 |
| Total Protein Foods | 6.9% | <0.01 |
| Seafood and Plant Proteins | 2.6% | <0.01 |
| Fatty Acids | 1.0% | <0.001 |
| **Moderation** |  |  |
| Refined Grains | 5.0% | <0.01 |
| Sodium | 3.5% | <0.01 |
| Added Sugars | 4.6% | 0.64 |
| Saturated Fats | 1.3% | 0.19 |

^1^*P*-value reflects the level of significance for macronutrients as a three-dimensional smooth term for the adequacy and moderation components of the Healthy Eating Index. Percentage of deviance explained (*Dev Exp.*) is shown for the entire model. Models were adjusted for age, household income, race/ethnicity, education level, smoking, alcohol, physical activity, BMI, and the Healthy Eating Index.

| **Supplemental Table 4.** Associations between Macronutrient Composition and Cardiometabolic Health: BMI Stratification Sensitivity in Females^1^ | | | | | | |
| --- | --- | --- | --- | --- | --- | --- |
| **Underweight and Healthy Weight Adults (n = 5,329)** | | | | | | |
| **Outcome** | **Model^1^** | | **Model^2^** | | **Model^3^** | |
|  | *Dev Exp.* | *P* | *Dev Exp.* | *P* | *Dev Exp.* | *P* |
| Triglycerides (mg/dL): n = 2,511 | 12.9% | <0.01 | 14.4% | <0.01 | 15.6% | <0.01 |
| Total Cholesterol (mg/dL): n = 4,678 | 14.5% | 0.78 | 14.9% | 0.81 | 15.0% | 0.83 |
| LDL Cholesterol (mg/dL): n = 2,450 | 10.8% | 0.45 | 11.2% | 0.50 | 11.7% | 0.51 |
| HDL Cholesterol (mg/dL): n = 5,006 | 4.8% | 0.01 | 6.2% | 0.03 | 7.7% | 0.04 |
| Glucose (mg/dL): n = 2,546 | 16.4% | 0.35 | 16.6% | 0.67 | 16.8% | 0.37 |
| Insulin (uU/mL): n = 2,485 | 3.9% | 0.15 | 4.2% | 0.21 | 5.6% | 0.14 |
| OGTT (mg/dL): n = 1,259 | 18.8% | 0.33 | 19.6% | 0.37 | 21.5% | 0.31 |
| HbA1c (%): n = 3,720 | 22.2% | 0.88 | 22.7% | 0.88 | 23.2% | 0.89 |
| Systolic Blood Pressure (mm/Hg): n = 5,098 | 44.8% | 0.16 | 45.0% | 0.19 | 45.2% | 0.16 |
| Diastolic Blood Pressure (mm/Hg): n = 5,062 | 9.4% | 0.84 | 9.9% | 0.75 | 10.1% | 0.80 |
| Body Fat (%): n = 713 | 2.2% | 0.94 | 2.2% | 0.94 | 5.2% | 0.96 |
| **Overweight and Obese Adults (n = 12,329)** | | | | | |  |
| Triglycerides (mg/dL): n = 5,414 | 4.0% | 0.41 | 6.2% | 0.03 | 7.9% | 0.60 |
| Total Cholesterol (mg/dL): n = 10,350 | 4.1% | 0.79 | 4.1% | 0.81 | 4.3% | 0.83 |
| LDL Cholesterol (mg/dL): n = 5,193 | 2.8% | 0.35 | 2.8% | 0.35 | 3.2% | 0.37 |
| HDL Cholesterol (mg/dL): n = 11,094 | 3.4% | <0.01 | 4.1% | <0.01 | 5.1% | <0.01 |
| Glucose (mg/dL): n = 5,480 | 12.2% | 0.55 | 12.5% | 0.54 | 12.8% | 0.50 |
| Insulin (uU/mL): n = 5,351 | 2.0% | 0.72 | 2.2% | 0.77 | 3.0% | 0.83 |
| OGTT (mg/dL): n = 2,524 | 14.5% | 0.20 | 15.2% | 0.19 | 16.0% | 0.19 |
| HbA1c (%): n = 8,357 | 15.3% | 0.53 | 15.7% | 0.52 | 16.0% | 0.50 |
| Systolic Blood Pressure (mm/Hg): n = 11,257 | 30.4% | <0.01 | 30.9% | <0.01 | 31.3% | <0.01 |
| Diastolic Blood Pressure (mm/Hg): n = 11,197 | 9.6% | <0.01 | 10.0% | <0.01 | 10.2% | <0.01 |
| Body Fat (%): n = 1,222 | 3.7% | 0.59 | 4.7% | 0.59 | 5.8% | 0.24 |

^1^Table shows the model outputs for the association of macronutrient composition and cardiometabolic health in females and stratified by BMI (above and below 25 BMI). *P*-value reflects the level of significance for macronutrients as a three-dimensional smooth term for triglycerides, total cholesterol, Low-density lipoprotein (LDL) cholesterol; high-density lipoprotein (HDL) cholesterol; systolic blood pressure; diastolic blood pressure; body fat percentage; glucose; insulin; oral glucose tolerance test (OGTT); hemoglobin A1C (HbA1c). Percentage of deviance explained (*Dev Exp.*) is shown for the entire model.

*Model^1^: Adjusted for Age, Household Income*

*Model^2^: Adjustments as per model 1 + Race/Ethnicity + Education Level*

*Model^3^: Adjustments as per model 2 + Smoking + Alcohol Intake + Physical Activity + Healthy Eating Index*

| **Supplemental Table 5.** Associations between Macronutrient Composition and Cardiometabolic Health: BMI Stratification Sensitivity in Males^1^ | | | | | | |
| --- | --- | --- | --- | --- | --- | --- |
| **Underweight and Healthy Weight Adults (n = 4,418)** | | | | | | |
| **Outcome** | **Model^1^** | | **Model^2^** | | **Model^3^** | |
|  | *Dev Exp.* | *P* | *Dev Exp.* | *P* | *Dev Exp.* | *P* |
| Triglycerides (mg/dL): n = 2,041 | 3.1% | 0.98 | 3.6% | 0.99 | 4.3% | 0.96 |
| Total Cholesterol (mg/dL): n = 3,802 | 10.6% | 0.47 | 13.3% | 0.10 | 13.7% | 0.18 |
| LDL Cholesterol (mg/dL): n = 1,990 | 12.6% | 0.13 | 11.2% | 0.43 | 11.7% | 0.56 |
| HDL Cholesterol (mg/dL): n = 4,094 | 1.6% | 0.03 | 2.0% | 0.03 | 3.4% | 0.02 |
| Glucose (mg/dL): n =2,064 | 9.4% | 0.49 | 9.6% | 0.50 | 10.3% | 0.55 |
| Insulin (uU/mL): n = 2,014 | 1.9% | 0.12 | 1.9% | 0.12 | 3.0% | 0.12 |
| OGTT (mg/dL): n = 1,082 | 20.0% | 0.90 | 20.4% | 0.87 | 21.4% | 0.83 |
| HbA1c (%): n = 3,149 | 13.4% | 0.82 | 13.3% | 0.45 | 14.1% | 0.83 |
| Systolic Blood Pressure (mm/Hg): n = 4,287 | 23.3% | 0.03 | 23.6% | 0.04 | 24.0% | 0.03 |
| Diastolic Blood Pressure (mm/Hg): n = 4,266 | 10.4% | 0.09 | 11.1% | 0.10 | 11.6% | 0.13 |
| Body Fat (%): n = 626 | 4.3% | 0.55 | 4.6% | 0.50 | 6.1% | 0.51 |
| **Overweight and Obese Adults (n = 11,575)** | | | | | |  |
| Triglycerides (mg/dL): n = 5,121 | 2.9% | 0.04 | 4.6% | 0.08 | 5.1% | 0.07 |
| Total Cholesterol (mg/dL): n = 9,738 | 5.8% | <0.01 | 6.3% | 0.01 | 6.8% | 0.02 |
| LDL Cholesterol (mg/dL): n = 4,800 | 5.8% | 0.03 | 6.3% | 0.04 | 6.7% | 0.06 |
| HDL Cholesterol (mg/dL): n = 10,519 | 2.2% | 0.14 | 2.8% | 0.22 | 4.2% | 0.03 |
| Glucose (mg/dL): n = 5,151 | 8.0% | 0.14 | 8.2% | 0.11 | 8.7% | 0.15 |
| Insulin (uU/mL): n = 5,080 | 1.1% | 0.82 | 1.2% | 0.80 | 2.4% | 0.83 |
| OGTT (mg/dL): n = 2,639 | 13.3% | 0.71 | 13.8% | 0.80 | 14.5% | 0.85 |
| HbA1c (%): n = 8,173 | 11.2% | 0.56 | 11.5% | 0.60 | 11.7% | 0.59 |
| Systolic Blood Pressure (mm/Hg): n = 10,675 | 10.0% | 0.12 | 10.4% | 0.16 | 10.5% | 0.14 |
| Diastolic Blood Pressure (mm/Hg): n = 10,625 | 11.8% | 0.32 | 11.9% | 0.28 | 12.1% | 0.30 |
| Body Fat (%): n = 1,146 | 4.3% | 0.10 | 4.4% | 0.11 | 5.1% | 0.17 |

^1^Table shows the model outputs for the association of macronutrient composition and cardiometabolic health in males and stratified by BMI (above and below 25 BMI). *P*-value reflects the level of significance for macronutrients as a three-dimensional smooth term for triglycerides, total cholesterol, Low-density lipoprotein (LDL) cholesterol; high-density lipoprotein (HDL) cholesterol; systolic blood pressure; diastolic blood pressure; body fat percentage; glucose; insulin; oral glucose tolerance test (OGTT); hemoglobin A1C (HbA1c). Percentage of deviance explained (*Dev Exp.*) is shown for the entire model.

*Model^1^: Adjusted for Age, Household Income*

*Model^2^: Adjustments as per model 1 + Race/Ethnicity + Education Level*

*Model^3^: Adjustments as per model 2 + Smoking + Alcohol Intake + Physical Activity + Healthy Eating Index*

| **Supplemental Table 6.** Associations between Macronutrient Composition and Cardiometabolic Health: Age Stratification Sensitivity in Females^1^ | | | | | | |
| --- | --- | --- | --- | --- | --- | --- |
| **Younger Adults (n = 8,950)** | | | | | | |
| **Outcome** | **Model^1^** | | **Model^2^** | | **Model^3^** | |
|  | *Dev Exp.* | *P* | *Dev Exp.* | *P* | *Dev Exp.* | *P* |
| Triglycerides (mg/dL): n = 4,000 | 1.2% | 0.03 | 4.7% | 0.05 | 14.6% | 0.03 |
| Total Cholesterol (mg/dL): n = 7,759 | 0.5% | 0.26 | 0.8% | 0.22 | 4.1% | 0.26 |
| LDL Cholesterol (mg/dL): n =3,880 | 0.3% | 0.32 | 0.5% | 0.32 | 4.8% | 0.29 |
| HDL Cholesterol (mg/dL): n = 8,245 | 4.0% | <0.01 | 4.9% | <0.01 | 15.4% | <0.01 |
| Glucose (mg/dL): n = 4,046 | 1.2% | <0.01 | 1.6% | <0.01 | 9.0% | 0.03 |
| Insulin (uU/mL): n = 3,968 | 2.2% | 0.86 | 2.9% | 0.84 | 34.9% | 0.75 |
| OGTT (mg/dL): n = 1,994 | 1.6% | 0.52 | 3.1% | 0.56 | 13.2% | 0.74 |
| HbA1c (%): n = 6,072 | 1.1% | 0.77 | 1.6% | 0.73 | 9.7% | 0.28 |
| Systolic Blood Pressure (mm/Hg): n = 8,274 | 0.3% | 0.66 | 1.4% | 0.64 | 11.6% | 0.71 |
| Diastolic Blood Pressure (mm/Hg): n = 8,262 | 0.7% | <0.01 | 1.6% | <0.01 | 4.3% | <0.01 |
| Body Fat (%): n = 1,882 | 0.6% | 0.76 | 0.6% | 0.75 | 33.3% | 0.89 |
| **Older Adults (n = 8,738)** | | | | | |  |
| Triglycerides (mg/dL): n = 3,925 | 1.5% | 0.01 | 4.8% | 0.02 | 8.3% | <0.01 |
| Total Cholesterol (mg/dL): n = 7,269 | 0.6% | 0.18 | 0.8% | 0.14 | 2.6% | 0.23 |
| LDL Cholesterol (mg/dL): n = 3,763 | 0.4% | 0.75 | 0.5% | 0.76 | 1.9% | 0.86 |
| HDL Cholesterol (mg/dL): n = 7,855 | 2.8% | 0.27 | 4.5% | 0.38 | 13.6% | 0.31 |
| Glucose (mg/dL): n = 3,980 | 2.3% | 0.38 | 3.0% | 0.44 | 7.8% | 0.48 |
| Insulin (uU/mL): n = 3,868 | 1.8% | 0.05 | 2.2% | 0.09 | 27.6% | 0.18 |
| OGTT (mg/dL): n = 1,789 | 3.4% | 0.13 | 4.5% | 0.13 | 8.9% | 0.25 |
| HbA1c (%): n = 6,005 | 3.1% | 0.23 | 3.8% | 0.20 | 8.8% | 0.22 |
| Systolic Blood Pressure (mm/Hg): n = 8,081 | 2.9% | <0.01 | 3.6% | <0.01 | 4.9% | <0.01 |
| Diastolic Blood Pressure (mm/Hg): n = 7,997 | 0.4% | 0.14 | 0.6% | 0.09 | 1.2% | 0.19 |
| Body Fat (%): n = 53 | 44.7% | 0.15 | 44.7% | 0.18 | 66.0% | 0.69 |

^1^Table shows the model outputs for the association of macronutrient composition and cardiometabolic health in females and stratified by age (above and below the median age of 48 years). *P*-value reflects the level of significance for macronutrients as a three-dimensional smooth term for triglycerides, total cholesterol, Low-density lipoprotein (LDL) cholesterol; high-density lipoprotein (HDL) cholesterol; systolic blood pressure; diastolic blood pressure; body fat percentage; glucose; insulin; oral glucose tolerance test (OGTT); hemoglobin A1C (HbA1c). Percentage of deviance explained (*Dev Exp.*) is shown for the entire model.

*Model^1^: Adjusted for Household Income*

*Model^2^: Adjustments as per model 1 + Race/Ethnicity + Education Level*

*Model^3^: Adjustments as per model 2 + Smoking + Alcohol Intake + Physical Activity + BMI + Healthy Eating Index*

| **Supplemental Table 7.** Associations between Macronutrient Composition and Cardiometabolic Health: Age Stratification Sensitivity in Males^1^ | | | | | | |
| --- | --- | --- | --- | --- | --- | --- |
| **Younger Adults (n = 8,274)** | | | | | | |
| **Outcome** | **Model^1^** | | **Model^2^** | | **Model^3^** | |
|  | *Dev Exp.* | *P* | *Dev Exp.* | *P* | *Dev Exp.* | *P* |
| Triglycerides (mg/dL): n = 3,686 | 0.5% | 0.07 | 2.0% | 0.10 | 11.3% | 0.08 |
| Total Cholesterol (mg/dL): n = 7,017 | 0.5% | 0.16 | 1.3% | 0.18 | 7.1% | 0.14 |
| LDL Cholesterol (mg/dL): n = 3,484 | 0.7% | 0.26 | 1.2% | 0.32 | 6.3% | 0.31 |
| HDL Cholesterol (mg/dL): n = 7,556 | 0.5% | 0.03 | 1.8% | 0.08 | 15.1% | <0.01 |
| Glucose (mg/dL): n = 3,706 | 0.4% | 0.31 | 1.1% | 0.25 | 6.9% | 0.16 |
| Insulin (uU/mL): n = 3,653 | 0.3% | 0.34 | 1.2% | 0.39 | 37.8% | 0.28 |
| OGTT (mg/dL): n = 2,107 | 1.5% | 0.35 | 2.5% | 0.40 | 10.2% | 0.54 |
| HbA1c (%): n = 5,937 | 0.4% | 0.32 | 1.2% | 0.35 | 7.0% | 0.31 |
| Systolic Blood Pressure (mm/Hg): n = 7,743 | 0.1% | 0.35 | 0.5% | 0.39 | 7.9% | 0.28 |
| Diastolic Blood Pressure (mm/Hg): n = 7,733 | 0.6% | 0.27 | 1.0% | 0.26 | 7.1% | 0.16 |
| Body Fat (%): n = 1,772 | 1.0% | 0.18 | 1.2% | 0.18 | 20.0% | 0.67 |
| **Older Adults (n = 7,719)** | | | | | |  |
| Triglycerides (mg/dL): n = 3,476 | 0.6% | 0.45 | 3.6% | 0.66 | 9.5% | 0.61 |
| Total Cholesterol (mg/dL): n = 6,523 | 0.4% | <0.01 | 1.4% | <0.01 | 3.1% | <0.01 |
| LDL Cholesterol (mg/dL): n = 3,306 | 0.8% | 0.02 | 1.7% | 0.02 | 3.5% | 0.03 |
| HDL Cholesterol (mg/dL): n = 7,057 | 0.6% | 0.47 | 1.5% | 0.45 | 13.8% | 0.05 |
| Glucose (mg/dL): n = 3,509 | 0.6% | 0.06 | 1.0% | 0.05 | 5.2% | 0.30 |
| Insulin (uU/mL): n = 3,441 | 0.3% | 0.66 | 2.9% | 0.84 | 28.2% | 0.46 |
| OGTT (mg/dL): n = 1,614 | 0.9% | 0.58 | 1.3% | 0.56 | 7.2% | 0.61 |
| HbA1c (%): n = 5,385 | 0.6% | 0.65 | 1.1% | 0.62 | 6.1% | 0.41 |
| Systolic Blood Pressure (mm/Hg): n = 7,219 | 0.9% | <0.01 | 1.6% | <0.01 | 1.7% | <0.01 |
| Diastolic Blood Pressure (mm/Hg): n = 7,158 | 0.3% | 0.39 | 0.3% | 0.40 | 1.1% | 0.46 |
| Body Fat (%): n = 0 | ˗ | ˗ | ˗ | ˗ | ˗ | ˗ |

^1^Table shows the model outputs for the association of macronutrient composition and cardiometabolic health in males and stratified by age (above and below the median age of 50 years). *P*-value reflects the level of significance for macronutrients as a three-dimensional smooth term for triglycerides, total cholesterol, Low-density lipoprotein (LDL) cholesterol; high-density lipoprotein (HDL) cholesterol; systolic blood pressure; diastolic blood pressure; body fat percentage; glucose; insulin; oral glucose tolerance test (OGTT); hemoglobin A1C (HbA1c). Percentage of deviance explained (*Dev Exp.*) is shown for the entire model.

*Model^1^: Adjusted for Household Income*

*Model^2^: Adjustments as per model 1 + Race/Ethnicity + Education Level*

*Model^3^: Adjustments as per model 2 + Smoking + Alcohol Intake + Physical Activity + BMI + Healthy Eating Index*

| **Supplemental Table 8.** Associations between Macronutrient Composition and Cardiometabolic Health: Cardiometabolic Sensitivity in Females^1^ | | | | | | |
| --- | --- | --- | --- | --- | --- | --- |
| **Outcome** | **Model^1^** | | **Model^2^** | | **Model^3^** | |
|  | *Dev Exp.* | *P* | *Dev Exp.* | *P* | *Dev Exp.* | *P* |
| Triglycerides (mg/dL) | 5.7% | 0.09 | 9.0% | 0.21 | 16.4% | 0.08 |
| Total Cholesterol (mg/dL) | 12.2% | 0.47 | 12.4% | 0.46 | 14.0% | 0.47 |
| LDL Cholesterol (mg/dL) | 9.8% | 0.06 | 9.9% | 0.07 | 12.4% | 0.06 |
| HDL Cholesterol (mg/dL) | 4.5% | <0.01 | 5.9% | <0.01 | 15.4% | <0.01 |
| Glucose (mg/dL) | 12.5% | 0.22 | 12.7% | 0.21 | 17.8% | 0.32 |
| Insulin (uU/mL) | 3.0% | 0.99 | 4.0% | 0.99 | 34.0% | 0.82 |
| OGTT (mg/dL) | 13.9% | 0.07 | 14.4% | 0.05 | 21.4% | 0.16 |
| HbA1c (%) | 16.4% | 0.82 | 16.8% | 0.78 | 21.2% | 0.63 |
| Systolic Blood Pressure (mm/Hg) | 33.5% | 0.01 | 33.9% | 0.01 | 37.4% | <0.01 |
| Diastolic Blood Pressure (mm/Hg) | 9.9% | 0.12 | 10.5% | 0.09 | 11.4% | 0.07 |
| Body Fat (%) | 2.1% | 0.88 | 2.1% | 0.87 | 4.6% | 0.86 |

^1^*P*-value reflects the level of significance for macronutrients as a three-dimensional smooth term for triglycerides (n = 5,077), total cholesterol (n = 9,488), Low-density lipoprotein (LDL) cholesterol (n = 4,928); high-density lipoprotein (HDL) cholesterol (n = 10,292); systolic blood pressure (n = 10,365); diastolic blood pressure (n = 10,330); body fat percentage (n = 1,684); glucose (n = 5,129); insulin (n = 5,032); oral glucose tolerance test (OGTT; n = 2,610); hemoglobin A1C (HbA1c; n = 7,509). Percentage of deviance explained (*Dev Exp.*) is shown for the entire model. Body fat percentage was not adjusted for BMI in Model^3^.

*Model^1^: Adjusted for Age, Household Income*

*Model^2^: Adjustments as per model 1 + Race/Ethnicity + Education Level*

*Model^3^: Adjustments as per model 2 + Smoking + Alcohol Intake + Physical Activity + BMI + Healthy Eating Index*

| **Supplemental Table 9.** Associations between Macronutrient Composition and Cardiometabolic Health: Cardiometabolic Sensitivity in Males^1^ | | | | | | |
| --- | --- | --- | --- | --- | --- | --- |
| **Outcome** | **Model^1^** | | **Model^2^** | | **Model^3^** | |
|  | *Dev Exp.* | *P* | *Dev Exp.* | *P* | *Dev Exp.* | *P* |
| Triglycerides (mg/dL) | 3.9% | 0.22 | 6.0% | 0.44 | 12.4% | 0.33 |
| Total Cholesterol (mg/dL) | 9.2% | 0.17 | 11.4% | 0.24 | 13.8% | 0.41 |
| LDL Cholesterol (mg/dL) | 10.8% | 0.26 | 9.7% | 0.18 | 12.0% | 0.28 |
| HDL Cholesterol (mg/dL) | 1.5% | 0.05 | 2.9% | 0.10 | 16.1% | 0.02 |
| Glucose (mg/dL) | 7.7% | 0.91 | 8.9% | 0.84 | 12.9% | 0.85 |
| Insulin (uU/mL) | 0.70% | 0.65 | 1.8% | 0.72 | 35.4% | 0.07 |
| OGTT (mg/dL) | 12.8% | 0.48 | 13.5% | 0.48 | 18.6% | 0.55 |
| HbA1c (%) | 9.3% | 0.44 | 9.9% | 0.45 | 13.8% | 0.44 |
| Systolic Blood Pressure (mm/Hg) | 14.4% | 0.18 | 14.75 | 0.22 | 18.1% | 0.24 |
| Diastolic Blood Pressure (mm/Hg) | 11.7% | <0.01 | 12.0% | <0.01 | 14.6% | <0.01 |
| Body Fat (%) | 4.0% | 0.18 | 4.4% | 0.17 | 5.3% | 0.32 |

^1^*P*-value reflects the level of significance for macronutrients as a three-dimensional smooth term for triglycerides (n = 4,605), total cholesterol (n = 8,538), Low-density lipoprotein (LDL) cholesterol (n = 4,387); high-density lipoprotein (HDL) cholesterol (n = 9,319); systolic blood pressure (n = 9,528); diastolic blood pressure (n = 9,500); body fat percentage (n = 3,291); glucose (n = 9,765); insulin (n = 9,602); oral glucose tolerance test (OGTT; n = 2,629); hemoglobin A1C (HbA1c; n = 7,145). Percentage of deviance explained (*Dev Exp.*) is shown for the entire model. Body fat percentage was not adjusted for BMI in Model^3^.

*Model^1^: Adjusted for Age, Household Income*

*Model^2^: Adjustments as per model 1 + Race/Ethnicity + Education Level*

*Model^3^: Adjustments as per model 2 + Smoking + Alcohol Intake + Physical Activity + BMI + Healthy Eating Index*

| **Supplemental Table 10.** Associations between Macronutrient Composition and Cardiometabolic Health: Pregnancy Sensitivity^1^ | | | | | | |
| --- | --- | --- | --- | --- | --- | --- |
| **Outcome** | **Model^1^** | | **Model^2^** | | **Model^3^** | |
|  | *Dev Exp.* | *P* | *Dev Exp.* | *P* | *Dev Exp.* | *P* |
| Triglycerides (mg/dL) | 10.1% | 0.07 | 12.9% | 0.17 | 17.7% | 0.03 |
| Total Cholesterol (mg/dL) | 10.4% | 0.64 | 10.5% | 0.63 | 11.4% | 0.77 |
| LDL Cholesterol (mg/dL) | 5.7% | 0.73 | 5.8% | 0.74 | 7.0% | 0.82 |
| HDL Cholesterol (mg/dL) | 4.7% | <0.01 | 6.2% | 0.01 | 16.8% | <0.01 |
| Glucose (mg/dL) | 11.2% | 0.28 | 11.7% | 0.30 | 16.7% | 0.37 |
| Insulin (uU/mL) | 2.8 | 0.18 | 3.4% | 0.29 | 32.1% | 0.36 |
| OGTT (mg/dL) | 16.3% | 0.02 | 16.9% | 0.03 | 22.6% | 0.07 |
| HbA1c (%) | 15.8% | 0.59 | 16.3% | 0.57 | 21.1% | 0.67 |
| Systolic Blood Pressure (mm/Hg) | 33.9% | <0.01 | 34.2% | <0.01 | 35.8% | <0.01 |
| Diastolic Blood Pressure (mm/Hg) | 8.7% | 0.02 | 9.0% | <0.01 | 9.3% | 0.01 |
| Body Fat (%) | 2.6% | 0.75 | 2.6% | 0.75 | 5.0% | 0.71 |

^1^*P*-value reflects the level of significance for macronutrients as a three-dimensional smooth term for triglycerides (n = 7,456), total cholesterol (n = 14,123), Low-density lipoprotein (LDL) cholesterol (n = 7,206); high-density lipoprotein (HDL) cholesterol (n = 15,178); systolic blood pressure (n = 15,410); diastolic blood pressure (n = 15,322); body fat percentage (n = 1,935); glucose (n = 7,553); insulin (n = 7,367); oral glucose tolerance test (OGTT; n = 3,764); hemoglobin A1C (HbA1c; n = 11,595). Percentage of deviance explained (*Dev Exp.*) is shown for the entire model. Body fat percentage was not adjusted for BMI in Model^3^.

*Model^1^: Adjusted for Age, Household Income*

*Model^2^: Adjustments as per model 1 + Race/Ethnicity + Education Level*

*Model^3^: Adjustments as per model 2 + Smoking + Alcohol Intake + Physical Activity + BMI + Healthy Eating Index*

| **Supplemental Table 11.** Associations between Macronutrient Composition and Cardiometabolic Health: Dietary Recall Sensitivity Including Only Individuals with Two Completed 24-hour Recalls in Females^1^ | | | | | | |
| --- | --- | --- | --- | --- | --- | --- |
| **Outcome** | **Model^1^** | | **Model^2^** | | **Model^3^** | |
|  | *Dev Exp.* | *P* | *Dev Exp.* | *P* | *Dev Exp.* | *P* |
| Triglycerides (mg/dL) | 5.9% | 0.75 | 9.0% | 0.73 | 15.7% | 0.67 |
| Total Cholesterol (mg/dL) | 6.6% | 0.72 | 6.8% | 0.70 | 7.9% | 0.65 |
| LDL Cholesterol (mg/dL) | 5.2% | 0.35 | 5.4% | 0.36 | 6.6% | 0.34 |
| HDL Cholesterol (mg/dL) | 4.0% | 0.59 | 5.3% | 0.61 | 15.2% | 0.44 |
| Glucose (mg/dL) | 13.2% | 0.87 | 13.9% | 0.86 | 12.9% | 0.85 |
| Insulin (uU/mL) | 3.6% | 0.40 | 4.2% | 0.43 | 35.4% | 0.07 |
| OGTT (mg/dL) | 16.2% | 0.04 | 17.0% | 0.05 | 18.6% | 0.55 |
| HbA1c (%) | 17.4% | 0.61 | 17.9% | 0.62 | 13.8% | 0.44 |
| Systolic Blood Pressure (mm/Hg) | 30.9% | 0.54 | 31.3% | 0.54 | 18.1% | 0.24 |
| Diastolic Blood Pressure (mm/Hg) | 9.0% | 0.02 | 9.4% | 0.02 | 14.6% | <0.01 |
| Body Fat (%) | 10.3% | 0.11 | 10.4% | 0.10 | 5.3% | 0.32 |

^1^*P*-value reflects the level of significance for macronutrients as a three-dimensional smooth term for triglycerides (n = 4,944), total cholesterol (n = 9,189), Low-density lipoprotein (LDL) cholesterol (n = 4,886); high-density lipoprotein (HDL) cholesterol (n = 10,127); systolic blood pressure (n = 10,279); diastolic blood pressure (n = 10,229); body fat percentage (n = 360); glucose (n = 4,995); insulin (n = 4,868); oral glucose tolerance test (OGTT; n = 3,352); hemoglobin A1C (HbA1c; n = 10,255). Percentage of deviance explained (*Dev Exp.*) is shown for the entire model. Body fat percentage was not sufficiently powered in Model^3^.

*Model^1^: Adjusted for Age, Household Income*

*Model^2^: Adjustments as per model 1 + Race/Ethnicity + Education Level*

*Model^3^: Adjustments as per model 2 + Smoking + Alcohol Intake + Physical Activity + BMI + Healthy Eating Index*

| **Supplemental Table 12.** Associations between Macronutrient Composition and Cardiometabolic Health: Dietary Recall Sensitivity Including Only Individuals with Two Completed 24-hour Recalls in Males^1^ | | | | | | |
| --- | --- | --- | --- | --- | --- | --- |
| **Outcome** | **Model^1^** | | **Model^2^** | | **Model^3^** | |
|  | *Dev Exp.* | *P* | *Dev Exp.* | *P* | *Dev Exp.* | *P* |
| Triglycerides (mg/dL) | 3.7% | 0.93 | 5.0% | 0.92 | 11.2% | 0.83 |
| Total Cholesterol (mg/dL) | 7.8% | 0.85 | 8.5% | 0.83 | 9.7% | 0.84 |
| LDL Cholesterol (mg/dL) | 7.0% | 0.81 | 7.6% | 0.84 | 8.8% | 0.76 |
| HDL Cholesterol (mg/dL) | 2.3% | 0.31 | 3.0% | 0.15 | 15.8% | 0.60 |
| Glucose (mg/dL) | 9.2% | 0.02 | 9.9% | 0.02 | 12.9% | 0.85 |
| Insulin (uU/mL) | 1.6% | 0.91 | 1.9% | 0.90 | 35.4% | 0.07 |
| OGTT (mg/dL) | 15.6% | 0.94 | 16.0% | 0.93 | 18.6% | 0.55 |
| HbA1c (%) | 12.2% | 0.87 | 12.7% | 0.89 | 13.8% | 0.44 |
| Systolic Blood Pressure (mm/Hg) | 11.8% | 0.80 | 12.1% | 0.79 | 18.1% | 0.24 |
| Diastolic Blood Pressure (mm/Hg) | 12.5% | 0.93 | 12.7% | 0.91 | 14.6% | <0.01 |
| Body Fat (%) | 4.5% | 0.84 | 5.4% | 0.79 | ─ | ─ |

^1^*P*-value reflects the level of significance for macronutrients as a three-dimensional smooth term for triglycerides (n = 4,476), total cholesterol (n = 8,348), Low-density lipoprotein (LDL) cholesterol (n = 4,349); high-density lipoprotein (HDL) cholesterol (n = 9,267); systolic blood pressure (n = 9,450); diastolic blood pressure (n = 9,425); body fat percentage (n = 386); glucose (n = 9,496); insulin (n = 4,436); oral glucose tolerance test (OGTT; n = 3,175); hemoglobin A1C (HbA1c; n = 9,330). Percentage of deviance explained (*Dev Exp.*) is shown for the entire model. Body fat percentage was not sufficiently powered in Model^3^.

*Model^1^: Adjusted for Age, Household Income*

*Model^2^: Adjustments as per model 1 + Race/Ethnicity + Education Level*

*Model^3^: Adjustments as per model 2 + Smoking + Alcohol Intake + Physical Activity + BMI + Healthy Eating Index*
